# A novel hotspot and rare somatic mutation p.*A138V,* at *TP53* is associated with poor survival of pancreatic ductal and periampullary adenocarcinoma patients

**DOI:** 10.1186/s10020-020-00183-1

**Published:** 2020-06-17

**Authors:** Gourab Saha, Richa Singh, Argha Mandal, Subrata Das, Esita Chattopadhyay, Prasun Panja, Paromita Roy, Navonil DeSarkar, Sumit Gulati, Supriyo Ghatak, Shibajyoti Ghosh, Sudeep Banerjee, Bidyut Roy, Saurabh Ghosh, Dipankar Chaudhuri, Neeraj Arora, Nidhan K. Biswas, Nilabja Sikdar

**Affiliations:** 1 Human Genetics Unit, Indian Statistical Institute, 203, B. T. Road, Kolkata, 700108 India; 2 Department of Biotechnology, Heritage Institute of Technology, Kolkata, India; 3 National Institute of Biomedical Genomics, Kalyani, West Bengal India; 4 Department of Pathology & Department of Gastrointestinal Surgery, Tata Medical Center, Rajarhat, Kolkata, India; 5 Division of Medical Oncology, Department of Medicine, University of Washington, Seattle, USA; 6 Department of Surgical Gastroenterology, Calcutta Medical Research Institute, Kolkata, India; 7 Department of General Surgery, Medical College and Hospital, Kolkata, India

**Keywords:** Pancreatic ductal adenocarcinoma, Periampullary adenocarcinoma, Novel somatic hotspot mutation, Frequently mutated genes, Next generation sequencing

## Abstract

**Background:**

Pancreatic Ductal Adenocarcinoma (PDAC) is a cancer of the exocrine pancreas and 5-year survival rates remain constant at 7%. Along with PDAC, Periampullary Adenocarcinoma (PAC) accounts for 0.5–2% of all gastrointestinal malignancies. Genomic observations were well concluded for PDAC and PACs in western countries but no reports are available from India till now.

**Methods:**

Targeted Next Generation Sequencing were performed in 8 (5 PDAC and 3 PAC) tumour normal pairs, using a panel of 412 cancer related genes. Primary findings were replicated in 85 tumour samples (31 PDAC and 54 PAC) using the Sanger sequencing. Mutations were also validated by ASPCR, RFLP, and Ion Torrent sequencing. IHC along with molecular dynamics and docking studies were performed for the *p.A138V* mutant of *TP53*. Key polymorphisms at *TP53* and its associated genes were genotyped by PCR-RFLP method and association with somatic mutations were evaluated. All survival analysis was done using the Kaplan-Meier survival method which revealed that the survival rates varied significantly depending on the somatic mutations the patients harboured.

**Results:**

Among the total 114 detected somatic mutations, *TP53* was the most frequently mutated (41%) gene, followed by *KRAS*, *SMAD4*, *CTNNB1*, and *ERBB3.* We identified a novel hotspot *TP53* mutation (*p.A138V*, in 17% of all patients). Low frequency of *KRAS* mutation (33%) was detected in these samples compared to patients from Western counties. Molecular Dynamics (MD) simulation and DNA-protein docking analysis predicted *p.A138V* to have oncogenic characteristics. Patients with *p.A138V* mutation showed poorer overall survival (*p = 0.01*). So, our finding highlights elevated prevalence of the p53*p.A138V* somatic mutation in PDAC and pancreatobiliary PAC patients.

**Conclusion:**

Detection of *p.A138V* somatic variant in *TP53* might serve as a prognostic marker to classify patients. It might also have a role in determining treatment regimes. In addition, low frequency of *KRAS* hotspot mutation mostly in Indian PDAC patient cohort indicates presence of other early drivers in malignant transformation.

## Introduction

Periampullary adenocarcinoma and PDAC account for 3.1% of all cancers and 4th leading cancer related deaths in Western countries (Waddell et al. 2015). They belong to a rare group of tumours which are often presented at an advanced stage and have poor prognosis. PDAC is tumour of pancreas which accounts for > 85% of all pancreatic head tumours (McGuire 2016). As the 5-year survival rate is constant at ~ 7%, it has been estimated that PDAC will become 2nd most common cause of cancer-related deaths by 2030 (https://seer.cancer.gov/) (Ying et al. 2016). The incidence of PDAC in India is 0.5–2.4 per 100,000 men and 0.2–1.8 per 100,000 women (Thapa 2015). Only one study from Eastern India (Kolkata cancer registry) showed 1.5% relative frequency in males and 1.2% in females for incidence of pancreatic cancer (Sen et al. 2002). Histologically, PACs are of two types; intestinal and pancreatobiliary subtypes (Chandrasegaram et al. 2016; Kumari et al. 2013). The morphology of pancreatobiliary tumours show close resemblance to pancreatic tumours. The incidence of PAC is low, approximately 0.5–2% of all gastrointestinal malignancies and 20% of all tumours of the extrahepatic biliary trees (Uomo 2014). Incidence of different subtypes of PAC varies among geographical locations (Chandrasegaram et al. 2016).

Extensive studies in Caucasian population showed *KRAS, TP53, CDKN2A*, and *SMAD4* genes as the early driver genes for PDAC (Hezel et al. 2006; Moore et al. 2003; Cowan and Maitra 2014). Using Next Generation Sequencing (NGS) studies, other driver genes and core signalling pathways have been identified for PDAC (Waddell et al. 2015; Bailey et al. 2016; Biankin et al. 2012; Witkiewicz et al. 2015). There are few studies for PACs which suggests a few other driver genes that were not reported in PDAC (Gingras et al. 2016; Sandhu et al. 2016). Though the management and treatment are similar for PDAC and PAC, compared to the 7% of 5 year survival of PDAC, PAC have better prognosis with 5 year survival in more than 30% (Chandrasegaram et al. 2016). PACs originating from the intestinal subtype have higher survival than pancreatobiliary subtype. Most of the studies proposed that cigarette smoking and alcohol consumption are the most important contributing lifestyle risk factors for PDAC in western world (Duell 2012). Although genomic landscapes of PDAC have been studied extensively in “western” patient populations, the genetic and epidemiological studies are limited in India. Appreciating the low incidence rate in India, compared to most part of the world, we hypothesised that the mutation profile of pancreatic cancer from western populations may differ from the Indian patients. In the present study, we performed NGS based DNA sequence analysis of selected cancer drivers and other related genes in a discovery cohort and re-sequenced a few functionally important regions by Sanger sequencing in a larger set of validation cohort to understand the recurrent mutation status of these genomic loci in PDAC and PAC patients from India.

## Methods

### Sample collection and selection

**Fig. 1:**
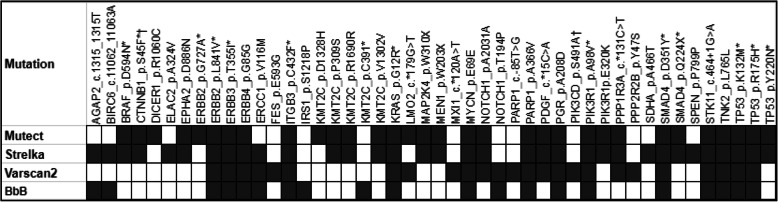
Somatic mutations identified by different variant caller. Somatic mutations identified by 4 different variant callers *Varscan, Strelka, Mutect and BbB* from 8 tumours. Black coloured boxes indicate mutation identified by variant caller and white boxes indicate “no mutation” identified by that variant caller. Mutations marked with “*” were selected for validation cohort. Mutations marked with “**†**” observed twice in patients

The study was approved by the Institutional Review Board (IRB) of Indian Statistical Institute, Kolkata and all involved hospitals. It was carried out following approved guidelines and informed consent of patients. The patient samples were collected from Tata Medical Center (TMC), Calcutta Medical Research Institute (CMRI), and Medical College and Hospital, Kolkata, India in between September 2013 to July 2017. All samples were confirmed by histopathologists of respective hospitals. Tumour tissue samples containing > 70% tumour content were selected for the study. A total of 93 patients comprising 36 PDAC, 28 PAC mixed subtype, 17 PAC intestinal subtype, and 12 PAC pancreatobiliary subtype were recruited in the study (S-Fig. 1). Eight patients (5 PDAC, and 3 PAC) were selected for NGS study and remaining 85 patients (31 PDAC, and 54 PAC) were selected for validation using Sanger sequencing method. Primary tumour, adjacent normal and blood samples were collected from each patient. Another set of 24 PDAC samples were used for validation of *KRAS* 12th codon mutation frequency.

### DNA extraction

DNA was extracted from tissue and blood samples using QIAGEN DNA extraction kit (DNeasy Blood and Tissue Kit, QIAGEN Inc., Germany).

### Library preparation and targeted next generation sequencing

Sequencing libraries were prepared from 1 μg of genomic DNA. DNA was fragmented using a Covaris LE220 Focused-ultra sonicator using factory settings for an average size of ~ 250 bp Sequence libraries were prepared using KAPA hyper-prep kit (KapaBiosystems, Wilmington, MA,USA) following end repair and A-tailing in a single-tube protocol. Indexed KAPA Hyper libraries were hybrid captured to NimbleGenSeqCap EZ custom probes (Roche) according to the manufacturer’s protocol. Custom oligonucleotide probes (Agilent’s) were designed to enrich targeted regions of interest, spanning 412 cancer genes (mostly exons) associated with different cancer types. Library size distributions were checked using Agilent Bioanalyzer and pooled library quantity was estimated using QubitFluorometer and Trinean DropSense96 spectrophotometer. Library DNA fragments immobilization and cluster amplification was performed on Illumina v4 flow cell using an IlluminacBot. Sequencing was performed using an IlluminaHiSeq 2500 in high-output 100-bp paired-end mode using v4 reagents (PE100). Sequence image data was analyzed using Illumina’s Real Time Analysis v1.18.66.3 software, followed by demultiplexing of indexed reads and generation of FASTQ files, using CASAVA1.8 pipeline (https://support.illumina.com).

### Data analysis and somatic variant calling

Raw sequence reads were mapped to human reference genome build (hg19) using BWA-mem. A total 10,98,38,875 reads were aligned from 8 pair samples with an average of 68,64,929 reads per library. The average attained depth of coverage for tumour samples were 137X and normal (blood) samples were 67x. Duplicate removal was performed using Picard (https://broadinstitute.github.io/picard/). Mapping quality was restricted to 40, and only uniquely mapped reads were kept. BAM files were generated using Samtools. GATK (https://software.broadinstitute.org/gatk/) was used to do local indel realignment and base quality recalibration. Somatic mutation calling was performed using four different orthogonal algorithms (frequency based, and heuristic threshold based), Varscan2 (Koboldt et al. 2012), MuTect (Cibulskis et al. 2013), STRELKA (Saunders et al. 2012) and BbB (India Project Team of the International Cancer Genome Consortium 2013) by analyzing variant containing reads from matched tumour and blood DNA data for every patient. Only those variants were considered that passed through filters of strand bias, total depth > 20, and population MAF < 0.01. Additional stringency of minimum 4 variant alleles at a variant site used to detect true somatic alteration. Variants were annotated using ANNOVAR (Wang et al. 2010). All variants were further manually curated using Integrative Genomics Viewer Version 2.3 (Broad Institute, Cambridge, MA).

### Analysis of Germline pathogenic mutation

DNA sequence reads generated from the blood tissues of 8 patients were also used to identify germline pathogenic variants. All variants were called using the Unifiedgenotyper tool in GATK packages. All variants with QV > 30 and depth of coverage greater than 10 were considered for further analysis. We have only focused on those mutations which are relatively rare in population databases like 1000 genomes or ExAC. We chose 0.1% population frequency cut-off to screen potential pathogenic variant candidates. We adopted ACMG recommended scoring as well as ClinVar annotation to annotate pathogenic or likely pathogenic variants in this small case series.

### Selection of region of interest and validation in a different patient cohort by sanger sequencing method

From the identified variants of NGS study, regions of interest were selected based on recurrent mutations, hotspot mutated regions and frequently mutated genes. These exons were then amplified by polymerase chain reaction (PCR) using specific primers (S-Tab. 2) and sequenced by Sanger DNA sequencing (ABI 3100 genetic analyzer) method in another 85 tumour samples comprising 31 PDAC and 54 PAC. The selected regions from NGS study were *TP53*_exon 5–8; NM:001126113, *SMAD4*_exon 6 & 9; NM:005359, *ERBB2*_exon 18 & 21; NM:004448, *ERBB3*_exon 9; NM:001982, *CTNNB1*_exon 3; NM:001904, *KRAS*_exon 2 and exon 3; NM:033360, *PIK3R1*_exon 2; NM:181523, *ITGB3*_exon 10; NM:000212, *BRAF*_exon 15; NM:004333, and *EPHA2*_exon 15; NM_004431. These somatic mutations were further confirmed for their absence in the corresponding blood or normal tissue samples.

### Mutation annotation and damaging property prediction analysis

The mutational damaging property of non-silent mutations were predicted (in silico) by PROVEAN (Choi and Chan 2015), SIFT (Sim et al. 2012) and Mutation assessor (Reva et al. 2011) tools (based on sequence homology and physical properties of amino acids). Public databases like The Cancer Genome Atlas (TCGA), Catalogue of Somatic Mutation in Cancer (COSMIC) (Forbes et al. 2017), *ClinVar* annotations were used to identify reported and novel variants.

### Verification and validation of somatic mutation by allele specific PCR

Mutations at *KRAS* and *TP53* (*p.A138V*), identified by Sanger sequencing and NGS methods were further validated and reconfirmed in corresponding samples by allele specific PCR (ASPCR) (Huang et al. 2015). Allele specific primers were designed against mutant sequence and PCR were performed to amplify DNA strands in the corresponding samples and visualized in 1% agarose gel. The primers (S-Tab. 2) were designed so that the last base of the forward primer changed as complementary to mutated allele. The mutations analyzed by this method were *KRAS: p.G12A, KRAS:p.G12V, KRAS:p.G12D, KRAS:p.G12R,* and *KRAS:p.Q61H. *PCR were performed in 10ul reaction volume composed of 2.0 mM MgCl_2_ and 0.25 U Taq Polymerase. A generalized initial denaturation for 5 min in 95 °C was followed by 35 cycles of denaturation for 15 s at 95 °C. Primer specific annealing temperatures were as follows: annealing for 25 s at 59 °C for *KRAS:p.G12A*, 58 °C for *KRAS:p.G12R*, 62.4 °C for *KRAS:p.G12V*, 61.9 °C for *KRAS:p.G12D*, 58 °C for *KRAS:p.Q61H* and 61.7 °C for *TP53:p.A138V.* We adopted a general PCR extension cycle time and temperature which was 40 s at 72 °C. End of 35 cycles were always followed by an extension allowance time of 5 mins at 72 °C. For *KRAS* G12 codon mutations, *KRAS*_G12*_R* reverse primer was used, for *KRAS* Q61 codon mutation *KRAS*_Q61_R reverse primer was used. For *TP53* A138 codon mutation, *TP53:p.A138V* mutant forward and mutant reverse primers were used with corresponding *TP53*_A138_R reverse, *TP53*_A138_F primers were used respectively. All primers used in this study are listed in S-Tab. 2.

### *KRAS* 12th codon mutation detection by two-step enriched-semi nested PCR

The presence of mutation in codon 12 of *KRAS* was detected using a Two-Step Enriched- Semi Nested PCR (Slebos et al. 2000). This technique involves two PCR amplifications, each followed by a restriction digestion. Using mismatch primer A (precise nucleotide change), and primer D a 197 bp fragment containing artificially generated restriction enzyme site (for *Bst*N1) at the 12^th^codon of *K-ras* gene was amplified in the first PCR. After digestion with *Bst*N1, the PCR products encoding mutant and normal sequences could be distinguished as 197 bp, 160 bp and 37 bp, band sizes respectively, in 2% agarose gel. A second PCR was done again using mismatched primer A and primer B (precise nucleotide change) to yield a 164 bp product using of the digestion resistant DNA fragment and undigested first PCR product (which was unenriched of mutant sequences). This time, PCR products with mutant and normal sequences could be distinguished as 147, 17 bp and 111, 36, 17 bp band sizes, respectively, after second *Bst*N1 digestion using unenriched mutant sequences from first PCR product. The mutant sequences reconfirmed with DNA fragments 147, 36 bp band sizes by second digestion with *Bst*N1 from enriched mutant sequences.

### *KRAS* mutation validation by ion torrent sequencing method

Six samples comprising 3 *KRAS* mutant and 3 *KRAS* wild type were selected for sequencing by Ion torrent PGM platform. DNA was extracted using QiagenGeneRead™ DNA FFPE kit (Qiagen, Hilden, Germany) following the manufacturer’s instructions. The NGS assay was performed with 10 ng of input DNA using the Ion Ampliseq Cancer Hotspot panel v2 [CHPv2] (Ampliseq, Life Technologies) on the Ion torrent PGM platform. The Ion AmpliSeq Cancer Hotspot Panel v2 is designed to amplify 207 amplicons covering approximately 2800 COSMIC mutations from 50 oncogenes and tumour suppressor genes. The amplicons were then digested, barcoded and amplified using the Ion Ampliseq Library kit 2.0 and Ion Xpress barcode adapter’s kit (Life technologies) according to the manufacturer’s instructions. The library was prepared using the Ion Ampliseq Library kit 2.0 with some modifications (Cycling was performed for 22 cycles for FFPE in a Veriti Thermal cycler and the reaction was reduced to half). The library was then quantified using the Ion Taqman quantitation kit (Life technologies). All the libraries were pooled in equimolar concentrations (100pM). The emulsion PCR was performed using the High Q view OT2 kit (Life technologies). The template ISP were enriched, loaded on a 318 chip and sequenced on a PGM sequencer with the Ion PGM High Q view sequencing 200 kit v2 according to the manufacturer’s instructions. Here we emphasized only on *KRAS* gene to validate mutations identified by other methods.

### Data analysis for the ion torrent sequencing data

The sequencing reads were aligned to hg19 using Torrent Suite 3.4.2. The sequences were analysed using variant calling software (Ion Reporter Annotate variants version 5.4, 5.6 and 5.10) caller to identify variants relevant to the clinical indication. The raw data was analysed using the Ion Browser Torrent Suite platform 5.05, 5.6 and 5.10 (Life technologies). Cases for which the number of mapped reads was < 100,000 and the average base coverage was <100X was rejected and repeated from library amplification. Mutations reported in the COSMIC (Sanger Institute Catalogue of Somatic Mutations in Cancer) database (http://www.sanger.ac.uk/cosmic) were reported whereas silent or intronic mutations were excluded. We have already established 2% as the limit of detection for this panel.

### Molecular dynamics simulation using Desmond

Molecular dynamics (MDS) simulations of wild type and *p.A138V* mutant at p53 were performed. Desmond version 5.6 was used for the molecular dynamics simulation study.

#### Pre-processing of PDB file

The wild type complex structure for molecular dynamics simulation has been adapted from the crystal structure of p53 core domain in complex with DNA (PDB code:4HJE). The mutated structure (*p.A138V*) of the complex was generated using Maestro 11.8.012. The monomers of (i) wild type and (ii) mutant *TP53* DNA binding domain bound to DNA were prepared using the Protein preparation wizard for molecular dynamics simulation. In addition, twin operations of H-bond assignment at pH 7.0 and energy minimization to eliminate any unfavourable interaction between protein and solvent molecules restrained energy minimization of heavy atoms at a minimum 0.30 Å RMSD range were also done for both systems using the Protein preparation wizard.

#### Solvent system addition to pre-processed molecular complexes

Desmond System Builder (https://www.schrodinger.com/desmond) was used to solvate both molecular complexes explicitly with TIP3P solvent system in an orthorhombic water box. The boundaries of each solvent box were at an average of 10 Å distance from the protein complex in three dimensions.

#### Molecular dynamics simulation and analysis

Both solvated complexes were subjected to 300 ns molecular dynamics simulation with a 10 ps trajectory-recording interval using Desmond 5.6 (https://www.schrodinger.com/desmond) at constant temperature (300 K) and constant pressure (1.01325 bar). The model systems were relaxed before simulation. The force field that was used was OPLS-2005 (Optimized Parameters for liquid simulations force field-2005 version) (Banks et al. 2005). This force field calculates the collision diameter for mixed interactions as the geometric mean of the values for the two component atoms.

The simulation outputs were then used to analyse time dependent average RMSD and secondary structure elements (SSE) of the protein complexes. The average residual RMSF of Wild Type and mutant p53 was also analysed from the simulation data. All these analyses were done using the Simulation interaction diagram panel of Desmond. Using Desmond Molecular dynamics event analysis panel, intermolecular hydrogen bonding formation between protein and DNA during the entire simulation has been calculated. The averaged value of total potential energy of the protein (p53 DNA binding domain/DBD comprises 91–291 aa.) part was calculated using the Desmond Molecular dynamics event analysis panel from the p53 DBD-DNA complex structures of entire dynamics simulation. Desmond trajectory clustering was used to cluster the structural frames according to their RMSD. Backbone and side chain RMSD were used to prepare the RMSD matrix at frequency of 5.

### p53-DNA binding interaction analysis using Haddock

Docking experiments were performed using (i) wild type and (ii) *p.A138V* mutant p53 DNA binding domain monomer against DNA as target. The wild type monomer was prepared from the crystal structure of the complex [PDB Code: 4HJE] available in protein data bank using UCSF chimera (Pettersen et al. 2004). The mutant monomer was modelled through the Swiss Model web server using the wild type *TP53* (chain A of PDB code: 4HJE) as template. The DNA was prepared using 3D – DART web server (van Dijk and Bonvin 2009) for the docking purpose [coordinates of DNA are adopted from PDB code: 4HJE]. Haddock 2.2 web server Guru Interface (van Zundert et al. 2016) was used for macromolecular docking. Different docking parameters were utilized other than that of the default setting for optimization of the p53-DNA interaction and subsequent analysis. All hydrogen atoms were used for calculation of distance restraints and additional calculations for the effect of solvent on docking and de-solvation energy of the protein-DNA complex were done.

### Tissue microarraySpot study for p53 in tumour tissues

The tumour site, morphologic subtype, grade, tumour size, margin status, presence of lympho-vascular (LVI) and perineural invasion (PNI), lymph node ratio (LNR), extranodal extension and stage were assessed. The tumours were staged according to the criteria in the American Joint Committee on Cancer (AJCC) 7th edition. A tissue microarray (TMA) block was made with 30 cases (*TP53* mutant) using 3mm diameter cores. Immunohistochemistry with DO-7 clone of p53 antibody (mouse monoclonal, sc-47698) from Santa Cruz, CA, USA was performed on the BondMAX automated immunohistochemistry staining platform from Leica using the standard operating protocols. The tissue cores were then scored for p53 immunostaining. We recorded the percentage of cells which stained positively as well as the intensity of staining. The cases in which more than 70% of the tumour cells stained positive with either moderate or strong intensity (positive) or those with absent staining (null-pattern), were considered positive. Other staining patterns were termed equivocal, representing wild type staining (Kobel et al. 2016).

### *ERBB2/Her2-Neu* amplification detection by TaqMan DNA copy number assay

*ERBB2* amplification was assessed by real time PCR using TaqMan probe comparing tumour vs. normal tissue/blood DNA samples (Vincent-Chong et al. 2017). Copy number analysis for *ERBB2* was done on all 93 samples using TaqMan Copy Number Assay (Hs00817646_cn) (Applied Biosystems, CA, USA). For copy number variation, 2^-Δct^values of tumour and normal group were compared by Wilcoxon signed rank test to identify significant difference of fold change in *ERBB2* copy numbers in tumour and normal groups (R Packages, R Studio, ggPlot*2*). Relative quantification determined as 2^-ΔΔct^ was calculated for each of the samples to identify copy number change. Above 2 fold change was considered as copy number changes. (Detailed method is in supplemental section).

### Genotyping of key polymorphisms of *TP53* and its associated genes and analysis

In order to analyse association between *TP53* somatic mutations and germline polymorphisms, we genotyped key polymorphisms in *TP53* (Arg72Pro-rs1042522, PIN3 Ins16bp, Intron 6 *Msp*I-rs1625895)*, MDM2* (SNP 309-rs2279744)*, p21* (Codon 31-rs1801270*),* and *p73* (73 bp Del) by PCR-RFLP and/or PCR method. We choose logistic regression and multifactor dimensionality reduction (MDR) model to study polymorphisms interactions with somatic mutations in our patient samples. (Detailed method in supplemental section).

### Statistical analysis

#### Survival analysis

Overall Survival (OS) analysis was done by Kaplan-Meier estimator using SPSS Inc. (Version 16.0, Harvard University, MA, USA). OS was calculated from the date of pathologic diagnosis to the date of death or the date of the last confirmed contact. Survival curves were generated using the Kaplan-Meier method and assessed for statistically significant differences (*p < 0.05*) via the log rank test. We compared OS difference between patients having *p.A138V* mutation in *TP53* with patients who do not harbour this mutation and also with patients who do not harbour any *TP53* mutations. Overall survival were also compared between PDAC and PAC patients group, and separately between *ERBB2*/*Her2-neu* amplified and *ERBB2*/*Her2-neu* non amplified group to study effect of *ERBB2*/*Her2-neu *amplification in patient survival. In addition to that, to identify effect of SNPs in patient survival, we estimated OS difference between risk genotypes and non risk genotypes of the stated polymorphisms at *TP53* and other *TP53* associated genes. Here, we choose a three dimensional survival model for all the polymorphisms where we compared at least two loci containing the risk genotypes out of three loci vs. at least two loci containing the non risk genotypes out of three loci. The risk vs. non risk genotypes were assumed in dominant model.

#### Simulation analysis for targeted exome sequencing

To address the low sample size issue in our cohort, we designed a simulation study with patient datasets from the TCGA Pancreatic Ductal Adenocarcinoma – PDAC and Ampullary Carcinoma-AC project (Yachida et al. 2016; Cancer Genome Atlas Research Network 2017). In the simulation study, we randomly sampled data from 8 patients from the 150 TCGA-PDAC patients and 60 AC patients separately and quantified occurrences of non-silent somatic mutations in TCGA reported PDAC driver genes and AC driver genes from downloaded somatic variant data. This random selection based sampling study was iterated for 10,000 times to obtain the mutation frequency estimates.

## Results

### Patient characteristics

The mean age of the patients was 53±10.36. Twelve percent (*n* = 11) of the patients had pancreatitis history (Table 1, and S-Tab. 3). The frequencies of pancreatitis were almost similar between PDAC and PAC patients (45% vs. 55% respectively). According to 7^th^ edition of American Joint Committee on Cancer (AJCC) nomenclature on the stages (pathological) of the tumours were; IA 6% (*n* = 6), IB 20% (*n* = 19), IIA 14% (*n* = 13), IIB 54% (*n* = 50), III 4% (*n* = 4), and IV 1% (*n* = 1). Thirty five percent (*n* = 33) of the tumours were well differentiated adenocarcinoma (WDA), 41% (*n* = 38) were moderately differentiated adenocarcinoma (MDA), 13% (*n* = 12) were poorly differentiated adenocarcinoma (PDA), but grades were not available in the remaining patients (Table 1, and S-Tab. 3).

**Table 1:** Characteristics of Demography and Clinicopathological parameters of total patients

Total Patients Recruited in the Study	***n*** = 93
**Demography & Clinicopathological Characteristics**	
Age (Mean)	53 ± 10.36
Smoking Habbit	34% (*n* = 32)
Alcohol Habbit	18% (*n* = 17)
Pancreatitis	12% (*n* = 11)
**Tumour Types**	
PDAC	39% (*n* = 36)
PAC unknown subtype	30% (*n* = 28)
PAC Intestinal subtype	18% (*n* = 17)
PAC Pancreatobiliary subtype	13% (*n* = 12)
**Tumour Classification (7th AJCC)**	
Stage IA	6% (*n* = 6)
Stage IB	20% (*n* = 19)
Stage IIA	14% (*n* = 13)
Stage IIB	54% (*n* = 50)
Stage III	4% (*n* = 4)
Stage IV	1% (*n* = 1)
**Tumour Differentiation**	
Well differentiated	35% (*n* = 33)
Moderately differentiated	41% (*n* = 38)
Poorly differentiated	13% (*n* = 12)
Unidentified	11% (*n* = 10)
**Lymph Node**	
Present	58% (*n* = 54)
Absent	42% (*n* = 39)

### Landscape of somatic mutation in discovery cohort

**Fig. 2:**
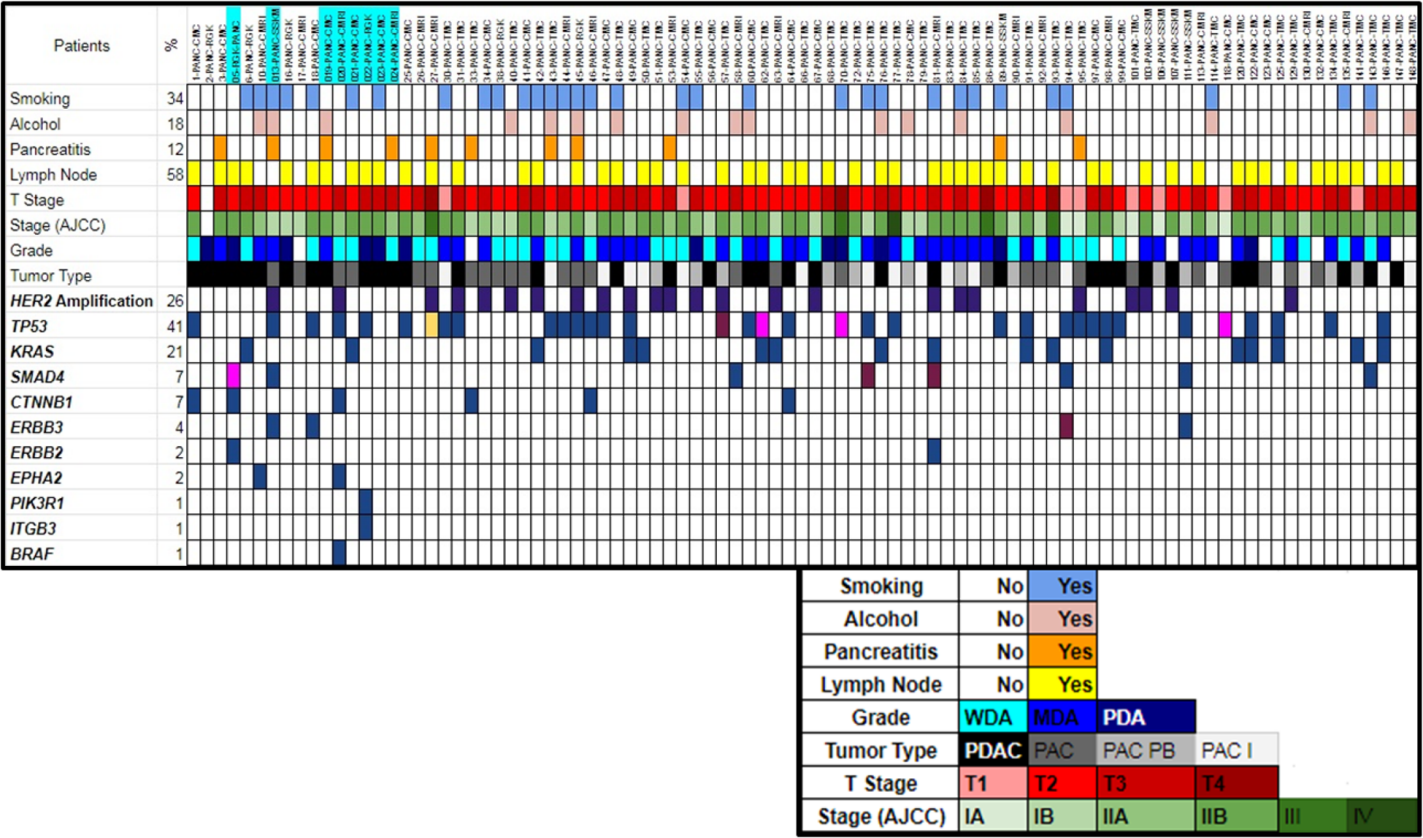
Patient characteristics and observed mutations in selected genes in total patient cohort(*n* = 93). Patient characteristics and observed mutations in selected genes in total patient cohorts (*n* = 93). Patient’s indicated with sky coloured boxes were the samples studied in targeted exome sequencing (NGS) method and remainingsamples were studied by Sanger sequencing method. The colours of the boxes in the demography and clinicopathological characteristics were explained in the small figure below the main figure. The blue boxes in the gene’s rows indicated presence of missense mutation, pink coloured boxes indicated presence of nonsense mutation, magenta coloured boxes indicated presence of silent mutation and yellow coloured boxes indicated presence of both missense and silent mutation in that gene for corresponding patient. In the *ERBB2* row, dark purple coloured boxed indicate > 2 fold *ERBB2/HER2* amplification detected in the corresponding patient

Using NGS method, we have identified total 48 somatic mutations from 8 patients in exons from 412 genes (Fig. 1). Seventy three percent (*n* = 35) of the variants were identified by at least two variant callers (S-Tab. 4). These 48 somatic mutations comprise 65% (*n* = 31) missense, 10% (*n* = 5) synonymous, 10% (*n* = 5) UTR, 8% (*n* = 4) nonsense, 4% (*n* = 2) *InDel*, and 2% (*n* = 1) splice site mutations. Genes which were mutated in 2 or > 2 samples are following: *KMT2C* (*n* = 5*), TP53* (*n* = 3), *SMAD4* (*n* = 2)*, CTNNB1* (*n* = 2)*, ERBB2* (*n* = 2)*, NOTCH1* (*n* = 2)*, PARP1* (*n* = 2)*, PIK3R1* (*n* = 2)*,* and *PIK3CD (n = 2)* genes (Fig. 1). All the 3 mutations in *TP53 *in 3 samples were identified in DNA binding domain. The mutation *p.S45F* in *CTNNB1* and *p.S491A* in *PIK3CD* was recurrent as observed in two different patients. Only one mutation, *p*.*G12R* was observed in *KRAS* in 1 patient only. Somatic mutations, identified by NGS, were also identified by Sanger sequencing method which showed clear peak for the mutant allele (S-Fig. 2).

Our additional simulation analysis with published datasets (see methods) showed that somatic mutations in all known PDAC driver genes (*KRAS, TP53, SMAD4, CDKN2A, GNAS, RNF43, ARID1A, TGFBR2, RREB1,* and *PBRM1*) and AC driver genes (*KRAS, TP53, APC, ELF3, SMAD4, CTNNB1,* and *MUC4*) can be reliably detected even in small subset of 8 patients when randomly selected (10,000 iterations) from large patient cohorts’ datasets (S-Tab. 5).

### Characteristics of Germline mutations

We performed the germline mutation analysis on NGS sequenced PAC and PDAC patients only. To highlight, we identified 2 of these 8 patients harbouring DNA repair pathway associated germline pathogenic/likely pathogenic mutations in *BRCA1*:NM_007297.3:c.4042C > T:*p*.Q1348* and in *ETS2*:NM_001256295.1:c.191G > A:p.W64*. We have also detected a common variant in *FGFR4* (NM_213647.2:c.1162G > A:p.*G388R***)** in 5 of the 8 patients, which is associated with cancer metastatic progression (Ezzat et al. 2013). In summation, we did not detect any second hit to these genes in the respective tumour samples.

### Landscape of somatic mutation in validation cohort

Sanger sequencing method identified 66 somatic mutations in 16 exons of 10 genes in the validation cohort (S-Tab. 6). Out of these 66 alterations, 58 were missense, 5 were silent, and 3 were stop-gain mutations, respectively. PCR amplification for *KRAS* exon2 could not performed in 10 patients (6 PDAC and 4 PAC) due to bad sequence quality of the PCR products in this region. Thus *KRAS* mutation frequency was calculated for 75 case series. In the validation study, 41% mutations were observed in selected exons of *TP53*, 21% mutations in *KRAS*, 7% mutations in *SMAD4,* 5% mutations in *CTNNB1*, 4% mutations in *ERBB3*, 1% mutation in *EPHA2*, and 1% mutation in *ERBB2/Her2-neu *gene. But, no mutations were identified in the exons of *BRAF*, *PIK3R1*, and *ITGB3* genes.

### Spectrum of somatic mutations in entire patient cohort

**Fig. 3:**
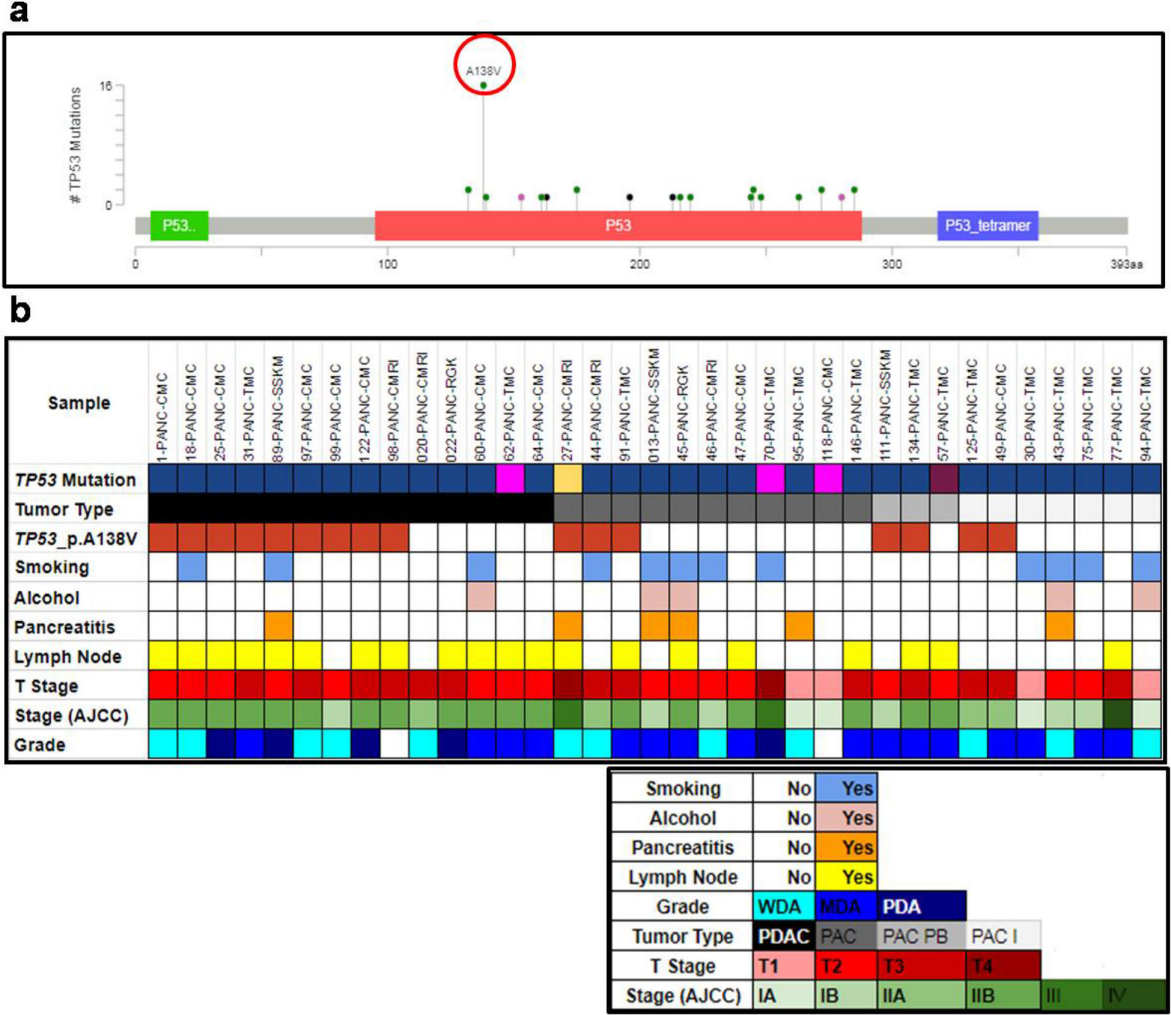
Mutational spectrum of *TP53* DNA binding domain. Mutational spectrum of TP53 DNA binding domain. **a** Frequency of *TP53* DNA binding domain mutations observed by *cBioPortal*. The red circle is the p.*A138V* mutation identified in 17% of all *TP53* mutations. **b** Patients (*n* = 35) with observed *TP53* mutations (i.e. 38% of total samples). In the sample row, sky coloured columns are the samples studied in targeted exome sequencing (NGS) and white columns are the samples studied in Sanger sequencing methods. The brown boxes in the *TP53 p*.*A138V* row indicate presence of p.*A138V* mutations in the samples. The colours of the boxes in the demography and clinicopathological characteristics were explained in the pictures below the main picture

**Fig. 4:**
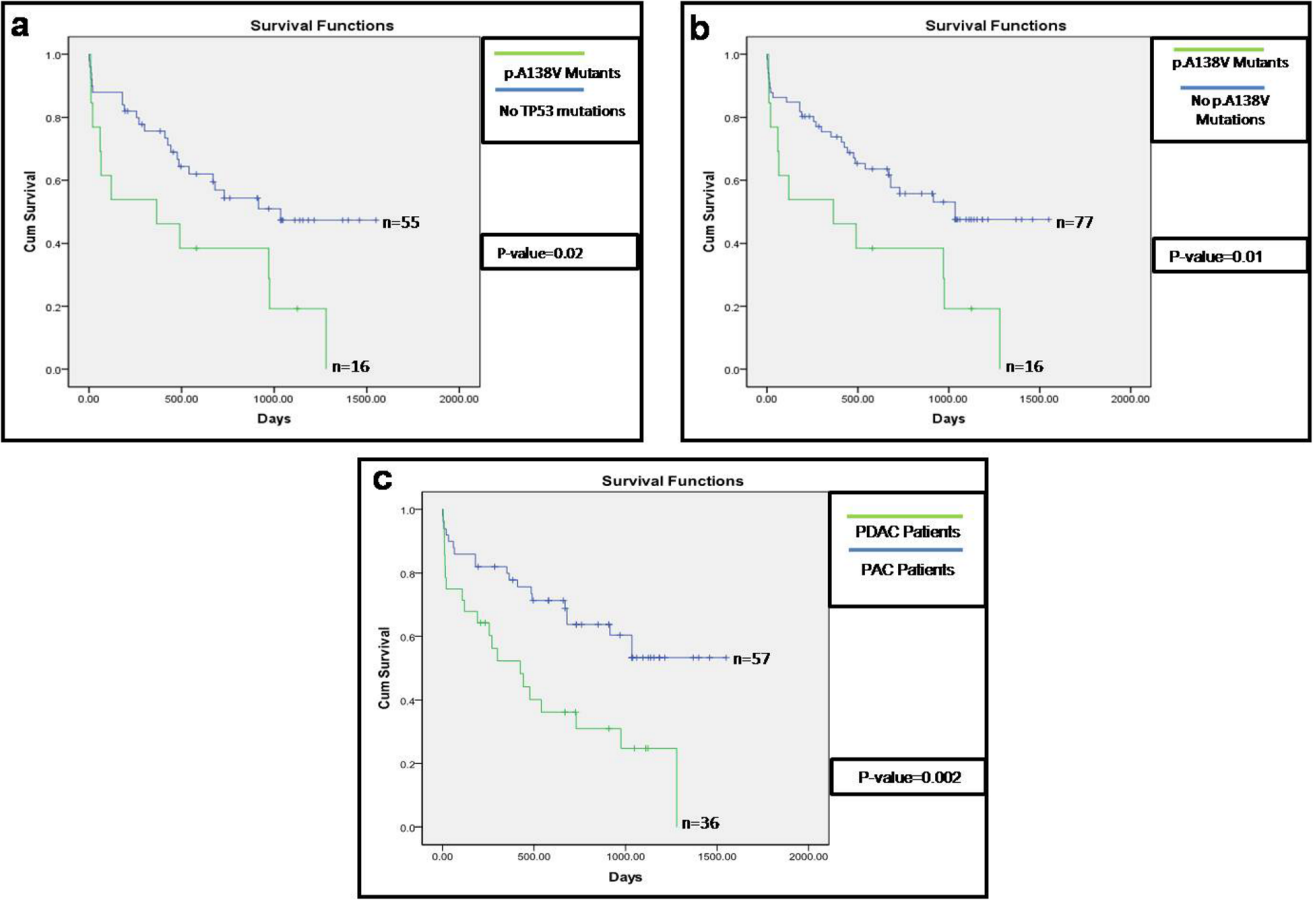
Survival graph observed by Kaplan-Meier estimator using SPSS. Overall survival comparison between different patient groups. **a** Kaplan-Meier overall survival analysis of patients with *TP53p.A138V* mutations and patients with “no *TP53”* mutations. **b** Kaplan-Meier survival analysis of patients with *TP53p.A138V* mutations and patients with “no *TP53p.A138V* mutations”. **c** Kaplan-Meier overall survival analysis of PDAC and PAC patients. Number of patients denoted by “n” in each of the survival curves

**Fig. 5:**
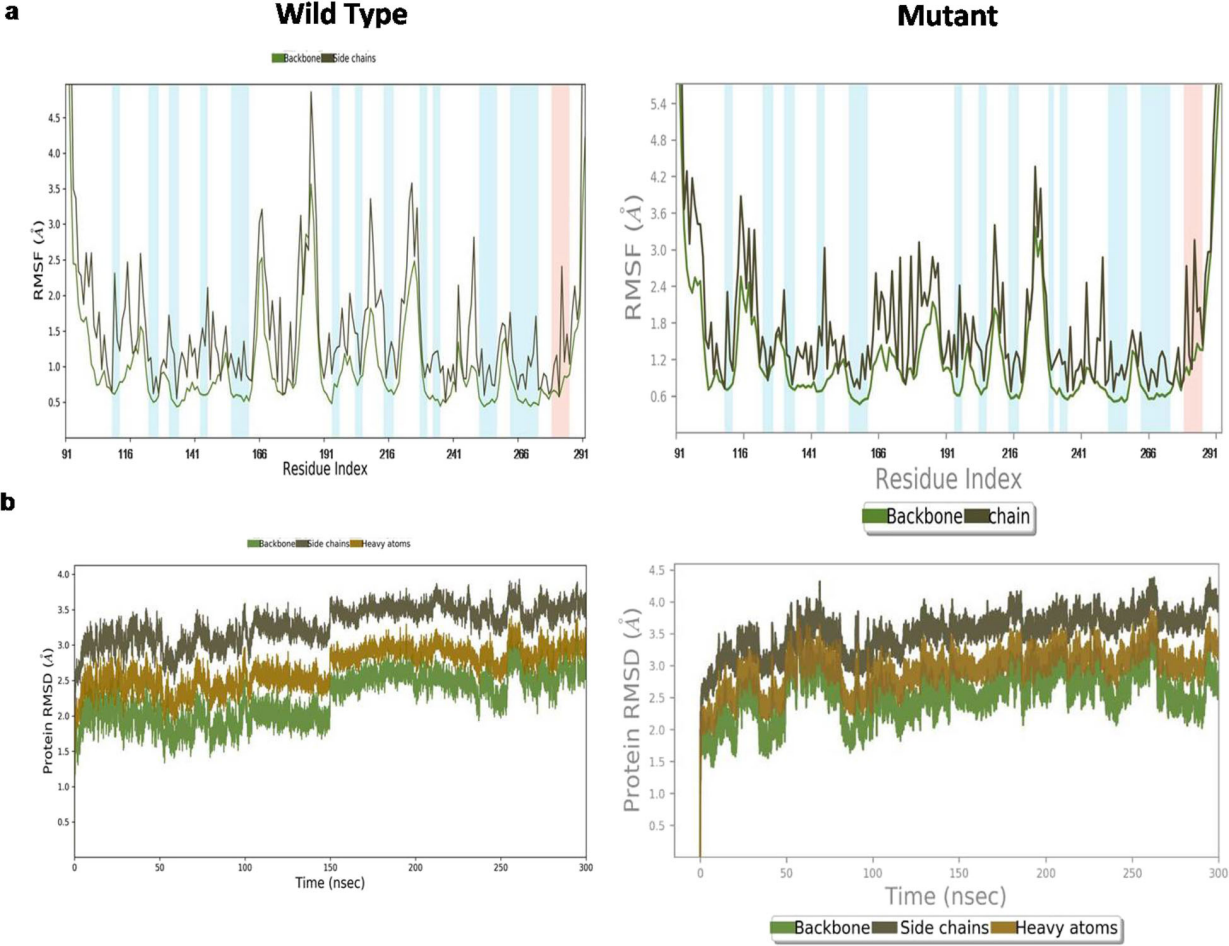
Molecular dynamics analysis of wt-type *TP53* and *mTP53 (p.A138V)*. Comparison of RMSF and RMSD plot between wild type and mutant (*p.A138V*) Tp53 protein. **a** The plot represents the time dependent RMSF plot of the *TP53* DNA binding domain of wild type and p.*A138V* mutant respectively. Residue 91–291of Tp53 protein represents the DNA binding domain/core domain of Tp53 crystal structure 4HJE.With respect to residues mapped in Tp53 protein, different loops and helixes were denoted like L1 loop (residues 112–124), L2 loop (residues 164–194) (interrupted by Helix H1 residues 163–178), and L3 loop (residues 239–251). **b** The plot represents the time dependent RMSD plot for backbone, side chains and heavy atoms of the *TP53* DNA binding domain of wild type (left) and *TP53p.A138V* mutant (right)

**Fig. 6:**
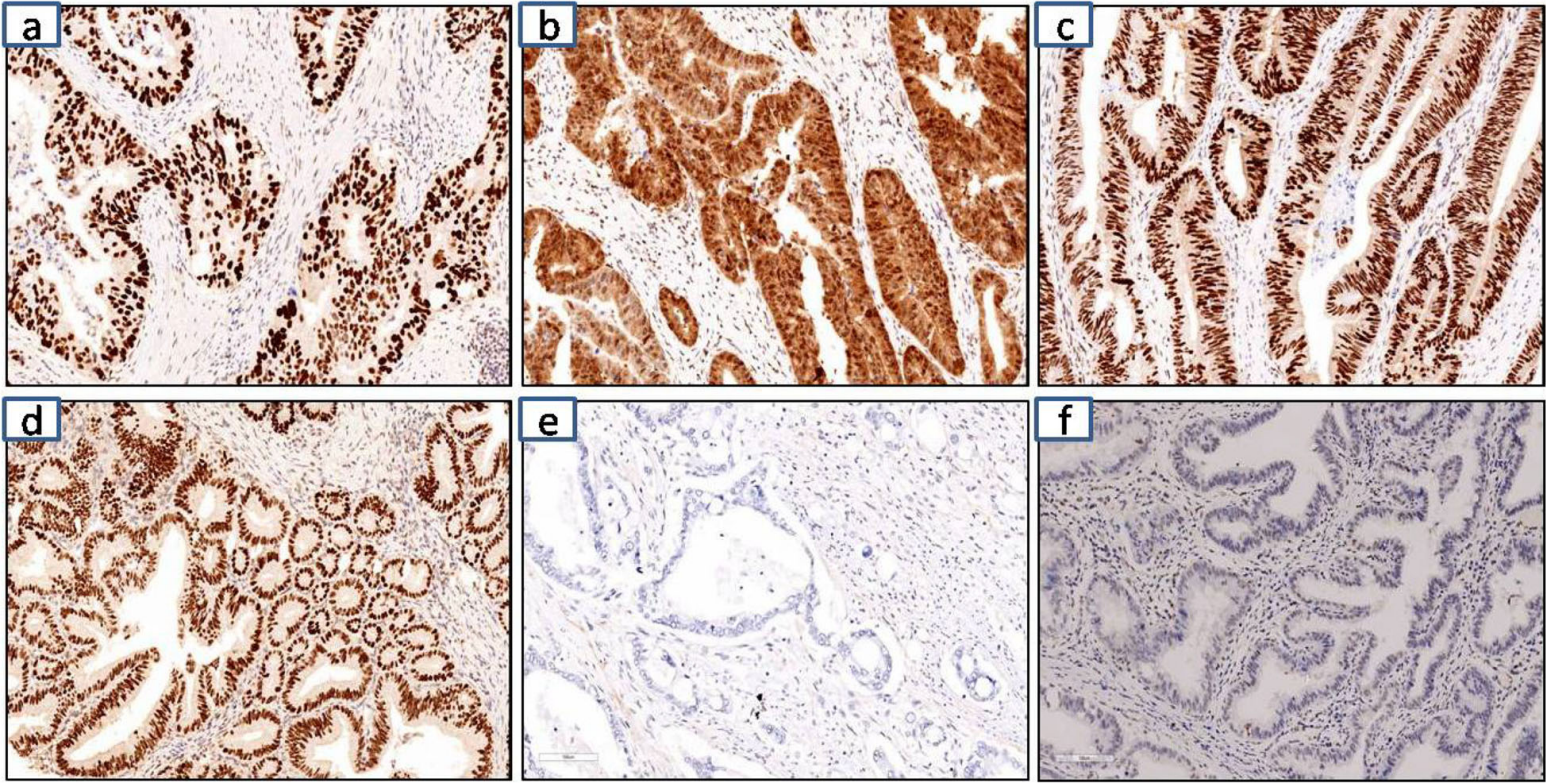
Tissue microarray analysis of Tp53 IHC staining. Staining is scored as follows; **a** & **b***TP53* p.A138V mutant tumours, **c***TP53* p.R175H mutant tumour, **d***TP53* p.E285K mutant tumour. All these above sections represents overexpression (OE) showing nuclear staining with strong intensity in > 70% tumour cell nuclei. **e** Tp53 null positive control tumour showing complete absence (CA) of expression in tumour cells. **f** Tp53 wild type tumour section showing nuclear staining with variable intensity in 1–80% of tumour cell nuclei or < 10% of tumour nuclei with strong intensity. All these section represents tissue microarray of pancreatic ductal and periampullary adenocarcinoma. All the above immunohistochemical snapshot was captured at 100 um from the respective TMA images

A total 114 somatic mutations comprising 112 point mutations and 2 deletions were identified in the total of 93 patient samples. Out of these 112 point mutations, 92 were missense, 7 were silent, 7 were nonsense, 5 were at UTR, and 1 was splice site alteration. The transition/transversion ratio was 2 with 67% transition and 33% transversion (S-Fig. 3). Among the mutations, 56% were identified in PAC patients (*n* = 64) whereas 44% mutations identified in PDAC patients (*n* = 50). *TP53* was identified as the most frequently (41%) mutated gene in our patient cohort (Fig. 2). Other recurrently mutated genes identified in our patient population were *KRAS* (21%), *SMAD4* (7%), *CTNNB1* (7%), and *ERBB3* (4%) (Fig. 2, S-Fig. 4). Whereas, less number of mutations were observed in the selected regions of *ERBB2* (3%), *EPHA2* (2%), *PIK3R1* (2%), *ITGB3* (1%), and *BRAF* (1%) genes in our patient population (Fig. 2). Recurrent alterations in these genes were *p.G12D* (*n* = 8), *p.G12A* (*n* = 3), *p.G12V* (*n* = 2), and *p.Q61H* (*n* = 2) in *KRAS*, *p*.*S45F/P* (*n* = 5) in *CTNNB1*, and *p*.*R361H/S* (*n* = 2) in *SMAD4* (S-Fig. 4). In our study, a total of 58 different types of missense mutations were identified. Seventy five percent (*n* = 44) of them were identified as probable damaging mutations by at least one prediction tools (*Provean, SIFT, and Mutationassessor*). In addition, 19 of the variants were annotated as pathogenic or likely pathogenic and 3 variants were reported as variants of uncertain significance in *ClinVar* database (S-Fig. 5). Sixty seven percent (*n* = 68) of the identified variants are also reported in COSMIC database. Whereas, 50% (*n* = 51) of the variants were reported in TCGA database. However, when we compared mutations identified in our PDAC patients (*n* = 36) with those in TCGA PDAC cohort (*n* = 185) only 13% (*n* = 6) of the mutations were found to be common (S-Fig. 6).

### Low frequency of *KRAS* mutations detected in PDAC and PAC cases

**Fig. 7:**
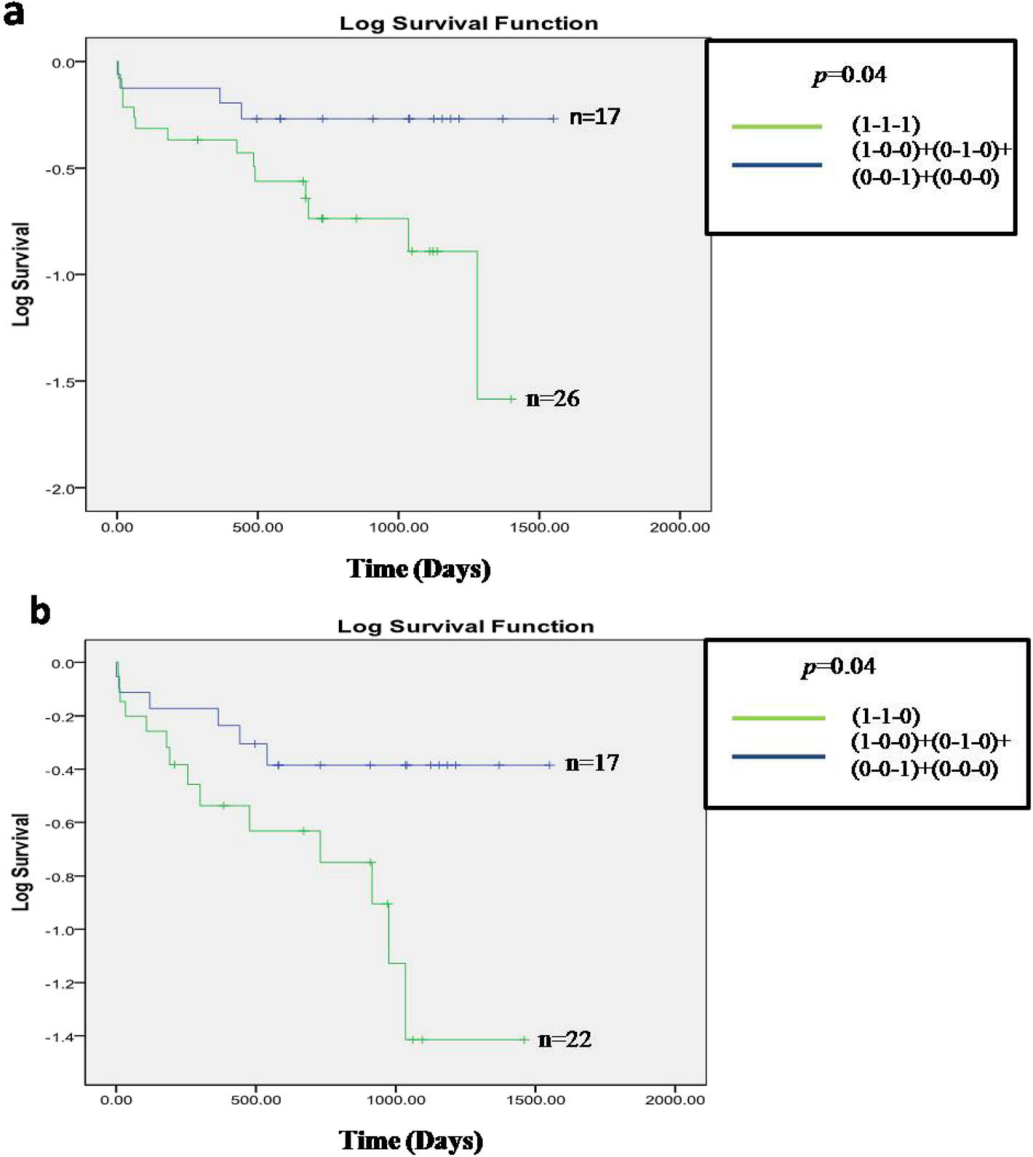
Survival analysis of combinational of polymorphisms in *TP53, MDM2* and *P73* genes. Overall survival comparison between patient’s group and combination of SNP markers of *TP53*, and its associated genes. In both picture (**a** & **b**) “1” indicates to patients containing risk genotypes and “0” indicates to patients containing non risk genotypes of all three loci. a. Kaplan-Meier OS analysis of patients with risk allele containing genotypes in *TP53* codon 72 (R/R, or R/P), *MDM2* SNP 309 (G/G, or T/G) and *P73* 73 bp deletion (D/D, or I/D) loci that denoted as () vs at least two loci with non risk genotype b. Kaplan-Meier OS analysis of patients with I/I genotypes (non risk) of *P73* (73 bp Indel) polymorphism in combination with risk genotypes of *TP53* codon 72 (R/R, or R/P), and *MDM2* SNP 309 (G/G, or T/G) loci vs all of the patients carrying at least two loci with non risk genotypes. Number of patients with respective genotypes denoted by “n” in each of the survival curves

*KRAS* is known to be very commonly mutated driver gene (90%) in pancreatic cancer (Cancer Genome Atlas Research Network 2017; Maitra and Hruban 2008). But, in this study, we found relatively lower frequency (i.e. 33%) of *KRAS* mutation in PDAC tumours. To revalidate this lower frequency, we adopted three independent mutation validation approaches. The mutations in *KRAS* and *p.A138V* mutant of *TP53* (*mTP53)* were further verified by allele specific PCR (ASPCR) (S-Fig. 7). Apart from Sanger sequencing method, *KRAS* 12th codon mutations were additionally validated by Two-Step Enriched-Nested PCR (S-Fig. 8). In case of PDAC, a total 10 alterations (8 in codon 12 and 2 in codon 61) were observed in 30 studied patient tumours. Whereas, a total of 7 alterations were identified in 53 PAC patient tumours (Table 2). In PDAC *KRAS* mutation study, 8 were identified by Sanger sequencing, 9 were validated by ASPCR and all 8 mutations at codon12 of *KRAS* were again validated by RFLP method (S-Fig. 9). In addition, 33% (8 out of 24) *KRAS* 12th codon mutations were identified in another independent cohort of 24 PDAC patients and validated by RFLP method. As we have observed *KRAS* has lower mutation frequency among Indian patient population compared to patient population from Western countries, we have also validated mutation frequencies by Ion Torrent method in 6 samples (3 *KRAS* mutants and 3 *KRAS* wildtype) to check the concordance with our Sanger sequencing method, ASPCR and RFLP results. All 3 *KRAS* mutants (G12D, G12C and Q61H), identified previously by other method, were also validated by Ion Torrent sequencing method.

**Table 2:** Identification of KRAS mutation in patient cohorts using different methods

Cohort	Total KRAS Mutation Identified (Codon 12 & 61)	Method	Tumour Type	***KRAS*** Mutation Frequency	Total ***KRAS*** mutation Frequency
**Discovery Cohort (*n*** **= 8)**	*N* = 1	NGS	PDAC (*n* = 5)	0%	12%
			PAC (*n* = 3)	33% (*n* = 1)	
**Validation Cohort (*n*** **= 85)/ (*n*** **= 75)**^**a**^	*N* = 16	Sanger Sequencing	PDAC (*n* = 25)	32%(*n* = 8)	19%
			PAC (*n* = 50)	14% (*n* = 6)	
		PCR-RFLP of 12th Codon	PDAC (*n* = 25)	32%(*n* = 8)	19%
			PAC (*n* = 50)	14% (*n* = 6)	
		ASPCR	PDAC (*n* = 25)	36%(*n* = 9)	20%
			PAC (*n* = 50)	12% (*n* = 6)	
**Independent Patient Cohort (*n*** **= 24)**	*N* = 8	PCR-RFLP of 12th Codon	PDAC (*n* = 24)	33% (*n* = 8)	33%

^a^In case of validation cohort PCR amplification of KRAS exon 2 for 10 samples could not performed due to technical error

### Novel hotspot somatic mutation at *TP53* is associated with poor prognosis

A total of 38 somatic alterations were identified in *TP53* DNA Binding Domain in all samples. Frequencies of these mutations among PAC and PDAC patients i.e. 25 in 57 PAC and 13 in 36 PDAC tumours, were not significantly different by Fisher exact test (*p = 0.51*). Thirty four of these 38 mutations were missense, 3 were nonsense, and 1 was silent mutation. Some of these *TP53* mutations were recurrent across tumours. We detected a novel hotspot missense mutation *p.A138V*, which was detected in 17% (*n* = 16) of all patients (Fig. 3a, and S-Fig. 10). All these *p.A138V* mutations were revalidated by ASPCR and also by Sanger re-sequencing of the ASPCR amplicon product (S-Fig. 7f). Surprisingly, the histological features of these 16 tumours resemble more close to PDAC and pancreatobiliary subtype of PAC. Briefly, 9 of 16 (56%) *p.A138V* mutations were in ductal tumours in origin, 2 (12.5%) were in pancreatobiliary subtype of PACs, 3 (19%) were in PACs mixed subtype and remaining 2 (12.5%) were intestinal type in origin (Fig. 3b). In TCGA database, this* p.A138V *mutation was reported to be in very low frequency {i.e. 0.04%, 4 out of 10,202 across Genomic Data Commons (GDC) database} but none of them were in pancreatic cancer. Analysis of this mutation, by all 3 functional prediction tools, identified it as a damaging missense mutation. The Kaplan-Meier survival analysis showed patients (*n* = 16) with the mutation at *p.A138V* in *TP53* has significantly poorer survival than patient (PAC and/or PDAC) having wild type variant (*p = 0.02*) (Fig. 4a). We also observed that patients with *p.A138V* mutation had a worse survival rate than the remaining ones (PAC and PDAC) (*p = 0.01*) in our patient cohort (Fig. 4b). It is to be noted that PDAC patients had a significantly poorer overall survival than PAC patients (*p = 0.002*) (Fig. 4c). The other detected recurrent *TP53* mutations in DBD were *p*.*E285K* (*n* = 2), *p*.*G245S* (*n* = 2), *p*.*R175H* (*n* = 2), and *p*.*V272M* (*n* = 2) (Fig. 3a).

### Molecular dynamics simulation analysis showed that *p.A138V* mutant behaves as a novel cancer mutant in *TP53*

We computed a residue specific differential Root Mean Square Fluctuation (RMSF) pattern in wild type and mutant p53 protein*.* At residue162–194 (L2 and helix H1 positions) we estimated more substantial fluctuations in wild type protein compared to its mutant type counterpart. This changed lability (i.e. conformational flexibility) may result in destabilization of the entire protein and also loss of affinity in the protein-DNA interaction in mutant**.** This was generally conferred from binding free energy. The fragment L2 (residues 162–194) and H1 (residues 163–178) creates a Zn^2+^ ion binding pocket with two residues 176C and 179H flanking it. The RMSF value suggests that the wild type protein has greater flexibility in that region of the protein as we observed the L2 and H1and other regions showing higher RMSF values in the wild type compared to the mutant.This illustrates that the *p.A138V* mutation at p53 loses its flexible conformation and becomes more rigid (Fig. 5a). This lower flexibility may affect the binding of the mutant to DNA and thereby reduce the tumour suppressor activity of the protein. Hence, next we performed DNA and protein molecule interaction by Haddock. The root mean square deviation (RMSD) for backbone, side chain and heavy atoms were analysed to study the convergence of the protein system for both wild type and *p.A138V TP53* mutant. In the RMSD plot, the native and mutant *p53* structure show a similar pattern of deviation from the start till 80 ns. Following this, the mutant structure showed a decrease in RMSD value in comparison to the native structure (upto 300 ns). Subsequently, the mutant *p.A138V* structure showed a smaller deviation till the end of simulations while the native structure indicated the same extent of deviation from the start till 100 ns and again from 150 ns to the end of the simulations (Fig. 5b).

In wild type, the SSE plot showed the presence of a small helix H1 (very less probable), a marginally more prominent existence of a small beta strand and a big loop L2. The SSE for mutant indicated small helix H1 which had become more prominent and strand had fully converted into a loop. During the simulation we noticed that the small helix H1 was predominant in the mutant protein. The trajectory clustering analysis using backbone and side chain RMSD resulted in 26 and 30 clusters for wild type and mutant *TP53* respectively (data not shown).

Total energy of p53 DBD was measured and statistically analysed during the simulation of the complexes. In our simulation, the average values of total potential energy (of only p53 DBD) were estimated as 12,383.123±75.461 and 12,470.351±97.519 kcal/mol respectively for wild type and mutant p53 DBD (data not shown). This result also supports the hypothesis that wild type DBD has greater stability than the mutant for the entire simulation period. The intermolecular hydrogen bonding significantly characterizes binding energy change between wild type and mutant *p53* DBD binding to DNA. Two types of intermolecular hydrogen bonds (O-H and N-H) are formed within the complexes in the 40 ns simulation timespan. The overall intermolecular hydrogen bonding statistics predicted greater number of intermolecular hydrogen bonds in wild type complex than in the mutant complex. During the simulation, number of overall intermolecular hydrogen bonding interactions varies with time in a range of 3–15 in wild type and 4–13 in mutant with the average of 8 and 7 for wild type and mutant respectively (S-Fig. 12). This supports the possibility of strong DBD-DNA binding in wild type compared to the mutant. Furthermore, this possibility is also supported by the count of N-H hydrogen bonds between DBD and DNA that are 9 and 5 for the mutant and wild type, respectively, throughout the simulation. However, within the wild type DBD-DNA dynamics, *R280* and *N288 *N-H bonds have 510 and 487 occurrence frequency respectively within 1000 frames. By comparison in mutant type DBD-DNA dynamics, *R248* is the only residue, which has an occurrence frequency of 203; occurrence frequency of 8 other N-H bonds is much less (data not shown). This observation also supports the conclusion that greater fluctuations of strong hydrogen bonding (N-H), lead to greater flexibility and instability of the mutant than that of the native protein. Additionally intermolecular hydrogen bond analysis from the trajectory visualization clearly highlighted a L2 loop which was highly active for mutant protein regarding intermolecular hydrogen bonding (residues *Q165* and *Q167)* with DNA. In contrast, in wild type protein, the L2 loop was not involved in intermolecular hydrogen bonding with DNA. It is thereby feasible that the increased RMSF of the L2 loop might have been due to the increased but less specific hydrogen bonding with DNA, which was not present in the wild type protein. In the wild type protein, the L1 loop was highly involved in intermolecular hydrogen bonding with *K120 *being the key residue forming hydrogen bond with DNA. However in the mutant protein the interaction of L1 loop with DNA had reduced, though *K120* was still the most interactive residue forming hydrogen bond. Alikely explanation of these results would be that a reduced RMSF of L1 loop in mutant protein was due to the reduced hydrogen bonding of L1 loop with DNA. Therefore mutation from alanine (A) to valine (V) at 138th position rendered L1 loop more rigid and L2 loop more flexible in comparison to the wild type. Consequently, the binding conformation of the protein might have been altered making the protein-DNA interaction more unstable.

### Interaction between DNA and protein (wild type vs. mutant p53) through molecular docking

Solvated Docking analysis with OPLS force field gave Haddock scores for docking with corresponding de-solvation energies calculated. Haddock scores are − 11,660.5+/−4.7 and − 11,538.8+/−31.7 (kcal/mol) for wild type p53 and mutant p53 protein respectively. Z-scores for both types were − 1.9. Buried Surface Area (BSA) for wild and mutant type are 1459.6+/−90.1 and 1230.9+/−78.8 Å^2^ respectively. The less negative values of the HADDOCK score of the *p.A138V* mutant p53 complexes (DNA and protein) suggested a lower affinity between biological partners (p53 protein-DNA) compared to that in native complex (S-Tab. 7). The BSA for wild type increased compared to mutant type indicating greater stability in the former. Thus, protein-DNA docking analysis and intermolecular hydrogen bonding patterns confirm that p53 protein has significantly decreased interaction with DNA due to the p53 DBD mutation at *p*.*A138V*, which may in turn affect function of p53 protein and inhibit tumour suppression.

### p53 nuclear accumulation is highly associated with presence of p53 missense mutation (*p.A138V*) in TMA of tumours

The sensitivity and specificity of the p53 TMA assay were examined in 2 pancreatic and periampullary tumours having *p.A138V* missense mutations, and 7 others known *TP53* missense DBD mutants included (*p.R175H, p.A161T, p.V216E, p.V272M, p.E285K (2) (*McGuire 2016*),* and *p. K132E,*). For tumours, p53 immunostaining was initially evaluated on TMA spots of IHC. We have adopted 3 tier scoring system to describe p53 staining: Overexpression (OE), Complete Absence (CA) or Wild Type (WT) in our TMA spot study. For negative control wild type and null type TP53 were taken for TMA analysis. The two cases of *p.A138V* mutant showed p53 nuclear accumulation/retention, and positive staining (> 70% of the tumour cells showing strong nuclear staining). For the p53 DBD mutations *p.V272M, p.R175H, p.E285K, p.K132E* also showed p53 nuclear accumulation and positive staining. Previously, *p.R175H *was well documented as a gain of function/oncogenic hotspot mutation. Thus all mutations showed positive or overexpression (OE) and this is most commonly associated with missense *TP53* mutants. In the negative control, we observed there is null type staining of p53 in the null type/complete absence tumour. We also detected equivocal p53 staining for wild type cases. In 5% of the cases, we have observed discordance between p53 missense mutation, and nuclear accumulation of p53 in TMA spot. It is noteworthy that in all of these cancers, heterogeneity of *TP53* expression could be observed, raising the possibility of focal *TP53* mutations in a tumour subclone that may not have been detected by the sequencing assays. High staining or positive staining also can be observed in case of frameshift, nonsense mutations and splice variants (Fig. 6).

### Association between combined genotypes of *TP53* and SNPs of its related genes with patient OS 

We have typed selected polymorphisms at *TP53* and its related genes (*p73, MDM2*, and *p21*) for all 93 patient samples. In combined genotype analysis we found in between *TP53* codon 72, *MDM2* SNP 309 and *p73InDel* 73 bp loci, patients carrying risk genotypes in these loci (R/R, or R/P + G/G, or T/G + D/D, or I/D) had significantly poorer OS (*p = 0.04*) compared to those carrying non risk genotypes in at least two loci (P/P + T/T + I/I) + (R/R, or R/P + T/T + I/I) + (P/P + G/T, or G/G + I/I) + (P/P+ T/T + D/D, or I/D) (Fig. 7a). In addition, we also observed in these three loci, patients containing I/I genotype of *p73InDel* 73 bp polymorphism with combination of risk genotype in the other two loci (*TP53* codon 72: R/R, or R/P + *MDM2* SNP 309: G/G, or T/G) also had poorer OS (*p = 0.04*) compared with the same combination like above (Fig. 7b). Furthermore, it is important to note that we could not identify any association between germline polymorphisms and *TP53* somatic mutations in DBD by both logistic regression and multifactor dimensionality reduction (MDR) model.

### *ERBB2/HER2-neu* amplification status in tumours

*ERBB2/Her2-neu* amplification was identified in 30% (17 out of 57) of the PAC and 19% (7 out of 36) in PDAC samples (Fig. 2, S-Fig. 13, and S-Tab. 8). Among the *ERBB2/Her2-neu* amplified patients, 50% (*n* = 12) of them found to carry mutation in the genes we studied, whereas remaining patients (*n* = 12) did not harbour mutation in the genes. However no significant survival differences were observed between amplified and non-amplified group. In addition, when fold change in expression between tumour and normal group were compared, no significant difference was observed (Wilcoxon signed rank test, *p = 0.91*). We have checked *ERBB2/Her2-neu* expression in few of the *ERBB2/Her2-neu* amplified tumour samples and observed overexpression in the tumour tissues compared to adjacent normal tissues, so amplification data followed the expression data (data not shown). Ideally the *ERBB2/Her2-neu* amplification results need to be validated by other methods like, in situ hybridization and immunohistochemistry (IHC), but it is limited by relevant tissue availability in our study.

## Discussion

Present study highlights mutational landscape of PAC and PDAC in Indian patients. In our targeted NGS discovery effort on 8 (PDAC and PAC tumours) and subsequent validation in another additional 85 more tumour tissues, we identified frequent mutations in genes like *TP53, KRAS, SMAD4, CTNNB1*, and *ERBB3.* Though, we have performed 8 paired (PDAC and PAC) samples, for targeted NGS studies, but after 10,000 times simulation with randomly taken 8 samples from databases showed similar sets of genes mutated in the diseased samples. This result confers the same genetic model predisposed if we increased the sample sizes in our discovery sample set. In multiple cancer types, all of these above 5 genes have been reported as driver genes with an array of frequently reported hotspot pathogenic mutations (Bailey et al. 2018). The *KMT2C* gene harbouring 5 somatic mutations in 3 of the 8 discovery tumours is one of the most frequently mutated genes among chromatin remodelling factors (Gonzalez-Perez et al. 2013) identified in PDAC tumours, but all detected mutations loci are spread over 56 exons of the gene, making the validation experiment design significantly harder to pursue.

Adenocarcinomas are known to exhibit relatively lower frequency of *G:C > T:A *transversion than *G:C > A:T* transition in PDAC and periampullary adenocarcinoma. Lower percentage of *G:C > T:A* transversion and higher percentage of *G:C > A:T* transition are consistent with our findings in pancreatic ductal and periampullary adenocarcinomas. Laboratory studies showed that the most common mutation caused by an alkylating agent is *G:C > A:T* transition (Greenblatt et al. 1994). Previously, it was reported substitution was 38% at *CpG* dinucleotides and 15% at *TpC* dinucleotides in PDAC cases (Jones et al. 2008). In our cases, only 13% of the substitutions were at *CpG* dinucleotides and 22% of the mutations were identified in *TpC* dinucleotides.

In our discovery cohort, frequency of *KRAS* mutations were 12% (S-Fig. 14), the result was consistent with our validation cohort (21%). *KRAS* however is more common (33%) among the PDAC patients. Interestingly, *KRAS* 12th codon mutation screening in another 24 PDAC independent cohort showed 33% mutations, which was consistent with our findings from previous patient cohort. In contrast, it was also known from earlier studies that *KRAS* was mutated in almost 70–90% of PDAC patients (Waters and Der 2018). Recent report from the TCGA datasets of 149 PDAC patients, showed mutation frequencies of major drivers *KRAS, TP53, SMAD4,* and *CDKN2A* are 93, 72, 32, and 30% respectively (Cancer Genome Atlas Research Network 2017). Disease progression model of PDAC confirmed that *KRAS* mutations are one of the earliest genetic events in normal duct to malignant transformation (Witkiewicz et al. 2015; Hruban et al. 2000; Eser et al. 2014). Ampullary tumours too harbour high frequency of *KRAS* mutations (40–50%) (Jones et al. 2008; Mikhitarian et al. 2014). In both PDAC and PAC, more than 90% of *KRAS* mutation occurs in 12thcodon (Prior et al. 2012; Bryant et al. 2014). So, in addition to Sanger sequencing and ASPCR approaches, we performed an RFLP based *KRAS* 12^th^ codon mutation detection assay in another 24 PDAC tumours as well as in the validation cohort. Among the 24 PDAC tumours, 8 (33%) found to harbour this *KRAS *12^th^ codon mutation, suggesting a congruence with the validation cohort results. Similar to previous reports, this study also identified almost 80% of all the *KRAS* mutation at 12^th^codon among all *KRAS* mutations in our PDAC tumours. Most of the European studies observed 72–83% of *KRAS* mutation whereas Korean studies observed only 47–52% *KRAS* mutation in PDAC. However, Japanese and Chinese pancreatic patients showed 94 and 71% *KRAS* mutation prevalence respectively (Song et al. 2000; Kwon et al. 2015). In pancreatobiliary malignancies the *KRAS* point mutation frequency known to vary between 75 and 100%, however *Oliveira-Cunha* et al. (2012), observed only 41% PAC patients had *KRAS* mutation in their primary tumours (Oliveira-Cunha et al. 2012). We observed a similar trend like colorectal cancer (CRC), in which an Indian cohort detected to have only 20.5% *KRAS *mutated patients, compared to~ 40% *KRAS* mutants in Western counterparts (Patil et al. 2013). Even in non small cell lung cancer (NSCLC), frequency of *KRAS* mutations differs between Caucasians and East Asian populations (25–50% and 5–15% respectively) (Choughule et al. 2014). A very recent cfDNA analysis revealed that *KRAS* mutation frequencies vary from 39 to 47% in PDAC patients from different studies (Gall et al. 2019). Till date, only one study from India reported 31% of PDAC patients harboured *KRAS* mutations using cfDNA based assay (Singh et al. 2015). Our data also supported the previous finding on Indian patients that *KRAS* mutation frequency varied from 30 to 33% in Indian PDAC cohort. Based on all previous observations on pancreatic, colorectal and NSCLC tumours in different world-wide population samples, we interpret that *KRAS* somatic mutations frequency is very much a population specific phenomenon which definitely is reminiscent of the fact that “one size fits all” population idea may not be valid here too. It is not very well understood why PDAC is associated exclusively with *KRAS* mutations in contrast to our observation. One explanation for the isoform specificity of *RAS* driven tumour formation could be explained by the fact that certain tissues are exposed to different carcinogens and environmental insults that result in mutations is specific to *RAS* genes. In addition to tissue based isoform specificity the frequency of different *KRAS* missense mutations can vary in different cancer types (Waters and Der 2018).

*Erb-B2* receptor tyrosine kinase 2 (*ERBB2/Her2-neu*) is a member of the *ErbB-RTK* family which comprises four closely related *RTKs*, such as *EGFR, ErbB2, ErbB3* and *ErbB4*. *ErbB2* dimerizes with itself or other *ErbB* members to activate downstream signalling pathway, such *as PI3K*-AKT. *ERBB2/Her2-neu* gene amplification occurs in a wide variety of human cancers (Ying et al. 2016). In our study, almost 19% of the PDAC patients showed *ERBB2/Her2-neu* amplification, whereas 30% of the PAC patients showed *ERBB2/Her2-neu* amplification in the tumour. Focal amplification of receptor tyrosine kinases (RTKs) such as *ERBB2*, *EGFR*, *MET*, and *FGFR1* have been already reported in PDAC (Assenat et al. 2015). Previously, *ERBB2* amplification also reported in ampullary adenocarcinoma ranging from 13 to 15% (Hechtman et al. 2015). The *ERBB2/Her2-neu* -targeted therapies, such as trastuzumab, lapatinib and pertuzumab, have showed improved outcomes in patients with *ERBB2/Her2-neu* amplification-positive cancers, and these drugs have been approved by the FDA against *ERBB2/Her2-neu*-positive gastric and breast cancers (Ying et al. 2016). In our study among the *ERBB2/Her2-neu* amplified group, 50% patients showed no mutation in any of the other most frequently mutated driver genes, indicating it can be a target of high therapeutic impact potential in *ERBB2/Her2-neu *positive PDAC and PACs.

The *WNT* signalling pathway has been previously reported to be implicated in PAC. Our study identified 7% mutations in *CTNNB1,* frequently at the 45th codon. Three of the mutations were identified in PDAC and remaining 3 mutations were identified in PAC cases. In case of PDAC, several studies (Bailey et al. 2016; Witkiewicz et al. 2015) reported mutations in *WNT* signalling pathway but not frequently in *CTNNB1* gene. On the other hand, in the case of periampullary tumours *WNT* signalling pathway was most frequently mutated in intestinal type than pancreatobiliary subtype (67% vs. 30%) (Gingras et al. 2016). Mutations in *CTNNB1* along with *APC*, *SOX9* and *FBXW7* were reported to be most frequent among periampullary tumours (Gingras et al. 2016). The increased rate of *CTNNB1* mutations and *WNT* alterations could greatly impact the choice of treatment since several *WNT* pathway targeted therapies are in the process of development.

In a previously published paper, the *TP53,*tumour suppressor gene (TSG) was reported to be inactivated in 40–75% of pancreatic tumours and among all cancers, 75–90% of somatic mutations were observed in the DBD of *TP53 (*Hainaut and Pfeifer 2016*)*. In our patient cohorts, *TP53* mutations were observed in 41% of the patients. Majority of the patients carrying *TP53* mutations were diagnosed in between stages IIA-IIB suggesting early inactivation of *TP53* during malignant transformation as in lung, head & neck and breast tumours. Interestingly, 3% of the patients showed more than one non-synonymous mutations in *TP53*. This finding suggests parallel evolution theory of tumour cells, where distinct lineages acquire mutations in the same cancer driver gene, leading to parallel subclonal expansion (Lawrence et al. 2013). The various kinds of *TP53* DBD mutations were identified in our patient cohort. Strikingly, *p.A138V* (17%) was the most frequent mutation among them (Fig. 3a). The Majority of *p.A138V* mutated cases were observed in PDAC (25%) but less in PAC (12%). In TCGA database, the frequency of *p*.*A138V* mutation in *mTP53* is 0.04% but no report in PDAC, though, this region is well conserved in different species.

Interestingly, it was found from the previous literature that *p*.*A138V* mutation in *TP53* was reported as both germline and somatic alteration in few studies (Lee et al. 2012). Similar to their observations we also detected *p.A138V TP53* as germline mutation in two of the patients (2%) (data not shown). However, we also screened this mutation in 130 unrelated healthy individuals collected from same population but none of them was found to carry this *p.A138V* mutation (data not shown). *In-silico* analysis with *Align-GVDV* predicted it to be likely deleterious (Petitjean et al. 2007). Earlier in-vitro studies suggested *p.A138V* is a temperature sensitive mutant (Milner and Medcalf 1990; Christgen et al. 2012). Recently, an *in-silico* study identified driver genes and mutations in cancers, by mutation clustering in three dimensional (3D) protein structures using mutations in 11,119 tumour samples across 41 tumour types with dataset contained 11,82,802 somatic missense mutations occurring in 10,25,590 residues in 18,100 genes. The p53 identified as the largest number of residues in 3D clusters. In 3038 rarely mutated residues in p53 that were clustered in 3D, *p.A138V/P/T* identified as one of the significant hotspot (*p = 0.02*) (http*:*//www.3dhotspots.org) (Gao et al. 2017). In a recent high throughput functional screening study for all possible DNA binding mutations in *TP53*, *p*.*A138V* hotspot mutation at *TP53* was found to have similar functional score (RFS > 0) as the well known *TP53* cancer hotspot positions (*p.R175H, p.G245S, p.R248Q, p.R249S, p.R273H* and *p.R282W*) (Kotler et al. 2018). A molecular docking simulation (MDS) study reported that *A138* codon of p53 is located at the interface of chain A and B of p53 dimer and DNA complex. The residue is conserved as well as surface exposed and considered as one of the binding hotspot amino acid loci (Ma et al. 2005). In addition to that, our MDS analysis (RMSF, SSE and Trajectory clustering) showed higher lability in the *p*.*A138V* mutant DBD compared to that of DBD of wild type p53. The predicted binding energy (as measured by docking score) suggests greater stability for wild type DBD of p53 compared to mutant one (S-Tab. 7). Overall, the mutant protein form is predicted to have a higher plasticity and higher degree of flexibility. All of these simulation derived characteristics showed consistent similarities with other well characterized hotspot drivers at *TP53* loci (*p.R175H, p.Y220C, p.G245S, p.R248Q, p.R249S, p.R273H,* and *p.R282W*) (Demir et al. 2011; Lepre et al. 2017). In addition, MDS also exhibited that *p.A138V* hotspot *TP53* mutant might have structural destabilization, and lesser affinity towards DNA molecule compared to the native form. Previous study reported similar results with *p.R273H* cancer hotspot mutation in p53 protein, losing its stability and becoming more rigid (Kamaraj and Bogaerts 2015). Subsequently, we performed a protein DNA docking analysis and intermolecular hydrogen bonding pattern simulation. As per this analysis, we have predicted *p.A138V* mutant form would have a significant affinity loss with DNA.

We also performed immunostaining of *p53  p.A138V* mutant cancer cases along with some other missense mutants. The majority of the *TP53* mutations are predominantly clustered in the DNA binding domain. Most of the mutations were associated with strong or intermediate staining of p53. In the MD simulation and docking study, we predicted that *p.A138V* has oncogenic properties like other known *TP53* hotspot mutants. Tissue Micro Array staining results also demonstrated that *p.A138V* has salient features like strong nuclear retention/stabilization in the nuclei, which is commonly observed in *TP53* hotspot/GOF mutants in cancer (Guedes et al. 2017). Additionally, survival analysis derived results showed this mutation associated with poor prognosis of the patients (*p = 0.01*). Previously, multitude of retrospective studies have associated abnormal p53 protein expression as well as somatic mutation with poor survival or lack of response to therapy. For cancers of the breast, head and neck, liver, hematopoietic, and lymphoid systems, a majority of studies showed an association of *TP53* mutations with poorer survival. However for cancers of the bladder, brain, lung, colon, esophagus, and ovary, several studies found no association of OS with *TP53* mutations (Robles and Harris 2010). In our study we found *p.A138V* mutated *TP53* was associated with poor OS of the patients. Furthermore, we also compared patient survival difference of *p.A138V mTP53* patients with other *TP53* hotspot mutants (such as *p.R175H, p.R248W, p.R248Q,* and *p.R282W*) of TCGA PDAC cohort data but no significant difference was observed among them (S- Fig. 15). All these evidences from previous observations and our findings concluded that *p.A138V* somatic alteration at *TP53* might have oncogenic role in development of PDAC and PAC in our patient population. We observed a very little overlap of *KRAS* and *TP53* pathogenic mutations in PDAC and PAC in these patients. It might be suggesting a possibility that a major fraction of PDAC and PAC carcinogenesis might have been facilitated by *TP53* mediated signalling pathways in a *KRAS* mutation independent manner. *Rowley* et al. (2011) demonstrated that inactivated *BRCA2* inhibited *Kras*^*G12D*^ associated pancreatic tumour but acted synergistically with disrupted *TP53* to promote pancreatic cancer in mice (Rowley et al. 2011). Similarly, detection of *KRAS* independent high frequency recurrent *TP53* mutation suggests the possibility of a newer pancreatic or pancreatobiliary carcinogenic transformation model for Indian patients. This could be due to differential lifestyle exposure and genetic composition making the studied Indian patients somewhat unique from their western counterparts. The OS analysis showed patients with pancreatic tumour surviving worse compared to patients with periampullary tumour. The previous reports also suggests the 5 year survival rate in PDAC is 6–8% whereas 30–50% in PAC patients (Chandrasegaram et al. 2016). Altogether, 50% of the all identified variants in our study found to be reported in TCGA database. But when we matched the mutation data of our PDAC patient cohort with mutation data of TCGA PDAC patient cohort, only 13% of the missense and nonsense mutations corroborated.

Different tumour types show different spectra of *TP53* mutations. The frequency of missense mutations also differs in different subclasses of tumours of the same organ. Our data indicated that *TP53 p.A138V* mutation is more frequent in PDAC and pancreatobiliary type of PAC. Above identified mutation is very uncommon in patients from other parts of the world. The new attractive therapeutic avenues point towards reactivation of some level of wild type function in mutant p53. A variety of compounds were recently identified, that might restore wild type p53 function. In addition to that, Short Interfering Mutant p53 Peptides (SIMPs) specific to the mutant p53 protein can restore the wildtype activity (Blandino and Di Agostino 2018; Muller and Vousden 2014). The identified hotspot mutation *p.A138V* could also be used as a therapeutic target like several other *TP53* hotspot mutations. Downstream pathways of *TP53 p.A138V* mutant may also be crucial for therapeutic intervention targets. Additionally, in the present study we observed *TP53 p.A138V* mutated patients have poorer survival suggesting this could be an useful prognostic biomarker. However, our data did not suggest any significant association between *KRAS* mutations and patient’s survival (data not shown). In contrast, other studies found significant association between *KRAS* mutation and poor prognosis (Kwon et al. 2015). *TP53* tumour mutations can be induced by environmental exposures that are distinctly different from patterns in other kind of cancers. This is what one might anticipate when different cancer types are associated with different risk factors that cause different mutation patterns in experimental systems. The codon 249 at *TP53* is highly mutated in hepato cellular carcinoma (HCC), due to exposures of aflatoxin B1, whereas in lung cancers, *TP53* mutations cluster at several codons, 157, 245, 248, and 273 due to exposures of tobacco smoke carcinogen metabolite, benzo-(a) pyrenediol-epoxide (BPDE). In melanoma, certain *TP53* mutations occur via exposures of sunlight (ultra-violet light) (Hollstein et al. 1998). The p53 protein has Cys3His1-typed zinc finger domain. Chronic toxicity from persistent exposure of heavy metal ions are weak mutagens in mammalian cells and has effects on zinc finger domain of p53 protein structure. Thus, impairment of p53-DNA binding capacity and inhibition of cell cycle arrest occurs (Koedrith and Seo 2011). With all previous facts explained by different group of researchers, we hypothesized that in case of our studied tumours, there might be specific exogenous carcinogens that have been conclusively linked with development of cancers. In our studied patient population, tobacco smoking habits and alcohol drinking habits were comparatively low (S-Tab. 3). However, most of the patients come from villages of West Bengal (one of the eastern states of India) and belong to low socioeconomic status group who may have had exposures of heavy metal particles through water intake, several kinds of occupational exposures and chemical exposures in agricultural field. Previously it was also reported that pancreatic cancers are associated with pesticide exposures, industrial chemicals, chlorinated hydrocarbons, solvents and nickel, organochlorines, and chromium (Andreotti and Silverman 2012).

There is a large body of evidence that suggest that p53 stress response pathway harbours inherited polymorphisms that affect p53 signalling in cells, resulting in differences in cancer risk and clinical outcome in humans. Previous studies inferred inherited variations in P53 pathway components may define patient populations in their abilities to produce apoptosis of cancer cells in response to DNA damage induced by chemotherapeutic agents (Grochola et al. 2010). While studying joint effect of SNPs of *TP53* and its associated genes, with patient survival, we found *TP53 *R72P polymorphism in combination with *MDM2* T309G and *p73* InDel 73 bp polymorphism played substantial roles in poor prognosis of PDAC and PAC. This suggests that the combined effect of ‘R’ allele in *TP53* codon 72, ‘G’ allele of *MDM2* SNP 309 and “D” of *p73* Indel 73 bp has adverse effect among all PDAC and PAC patients regardless of somatic *TP53* mutation. We took a closer look at *TP53* as we genotyped 3 well known cancer associated polymorphisms (R72P-rs1042522, PIN3 InDel 16bp, Intron 6 *Msp*I- rs1625895) in *TP53*. Haplotype analysis of these polymorphisms showed no significant differences in haplotypes frequency estimation between *TP53* mutant and non mutant group (S-Tab. 9). Additionally, we also investigated whether individual polymorphism or in combination of polymorphisms in *TP53* (R72P-rs1042522, PIN3 InDel 16 bp, Intron 6 *Msp*I- rs1625895), *p73* InDel (73 bp deletion), *p21* (codon 31- rs1801270*)*, and *MDM2* (SNP 309- rs2279744)*,* associated with mutant group. However, in both logistic regression and MDR model test showed no significant association, between risk SNP or in combination of risk SNPs with *TP53* somatic mutated patient group (S-Tab. 10 and 11). We could not correlate this specific combination of risk SNPs with somatically DBD mutated *TP53* patients (data not shown). To our knowledge, this novel finding is reported for the first time in PDAC and PAC disease groups. Similar to our result *Liu et.al* (2011) also showed the collective effect of *TP53*, *MDM2*, and *p73 *risk polymorphisms that have significant impact on prognosis of NSCLC patients (Liu et al. 2011).

Some limitations must be considered to interpret the data of our study. At first, we studied only selected cancer related genes so did not observe any alteration in these genes in 31 patients (33%), suggesting that different driver genes were involved in those patients. Those could have been better studied by whole exome/genome studies. Second, the sample size in the targeted study for NGS was only 8, which may have led to identification of less number of frequently mutated genes. Third, although we checked the tumour purity (> 70%) for almost all samples, we might have missed low frequency mutations (< 20%) (Rohlin et al. 2009) in samples as Sanger sequencing may not have identified such low frequency mutations. Fourth, the sample size for polymorphism and somatic mutation association study needed more samples for better result. Lastly, no satisfactory explanation could be given for low *KRAS* mutation frequency in Indian PDAC patients without studying a much larger cohort. It would be ideal in the future to validate the Indian PDAC patients’ *KRAS* mutation frequency in a statistically valid large Indian cohort.

## Conclusions

We, for the first time, revealed the mutational spectrum of some frequently mutated genes in PDAC and PAC patient cohort from India. We observed high frequency of a rare variant, *p.A138V *at *TP53* which could be considered as a novel hotspot cancer mutation. This may have oncogenic property as indicated by our experimental results (MDS and TMA studies) as well as a previous functional screening study. Our study identified *TP53* driven mechanism to be a dominant player over *KRAS* signalling networks, pointing to different environmental exposures in disparate geographical locations of Indian and Western countries. We identified a very low frequency of *KRAS* mutations compared to patients from Western countries and confirmed the data with 4 different experimental methods. This indicates the mutational landscape of frequently mutated genes in PDAC and PAC differs in our patient population with previous studies. In future, *p.A138V* hotspot oncogenic mutation may help clinicians for further patient stratification and therapeutic management to improve outcomes for this morbid diseases. To our knowledge, this is among the first studies to demonstrate that a specific *TP53* mutation is associated with poor prognosis of pancreatic cancer such as PDAC and PAC.

## Supplementary information

**Additional file 1: Fig. 1.** Hematoxilin and Eosin (H&E) staining of types of tumors. **a**. H&E staining of a PDAC tumor (left 10x magnification, right 20x magnification). **b**. H&E staining of an intestinal type of PAC tumor (left 10x magnification, right 20x magnification). **c**. H&E staining of a pancreatobiliary type of PAC tumor (left 10x magnification, right 20x magnification). **Fig. 2.** Validation of selected variants. Few of the somatic mutations identified in NGS study are being validated by Sanger sequencing method. Validated nucleotides are marked by vertical lines, in each pair upper one is for tumor DNA and lower one is for normal tissue or blood DNA. **Fig. 3.** Signature of somatic mutations identified in total patient cohort (*n* = 93). **a.** In the X axis different types of mutations is given and Y axis denoting frequency of types of mutations. **b.** Transition and transversion ration of all point mutations. **c.** In X axis six different signatures of point mutations is given and Y axis denoting the frequency of different signatures. **Fig. 4.** Frequency of mutations in other recurrently mutated genes in all patients. Different functional domains of proteins are indicated by different colours. The Y axis denotes frequency of mutations and X axis denotes mutations in different positions of the protein domains. Each vertical bar indicates mutation . Recurrent mutations are marked with red circle **a.***KRAS* mutations.**b**. *SMAD4* mutations. **c**. *CTNNB1* mutations. **Fig. 5.** Damaging and pathogenic variants of non synonymous mutations (*n* = 57). Damaging and pathogenic variants of non synonymous mutations (*n* = 57). The dark ass coloured boxes indicates mutations identified as damaging by different functional prediction tools *(Provean, SIFT, and Mutation Assessor)*. The white coloured boxes mean the tolerated mutations whereas light grey coloured boxes mean “unable to identify”. All the dark grey coloured boxes in the *ClinVar_Status* row indicate pathogenic or likely pathogenic variants and black coloured boxes are reported as uncertain significance in *ClinVar* database. **Fig. 6.** Comparison of reported and novel variants observed in this study with those reported in TCGA and Cosmic database. These are the total mutations identified by both NGS and Sanger sequencing method. The blue coloured boxes in sample row are PAC mixed samples, Sky coloured boxes are PAC intestinal samples, Grey coloured boxes are PAC pancreatobiliary samples and yellow coloured boxes are PDAC samples. The red colouredboxes in COSMIC and TCGA row are the reported mutation in these databases. The boxes marked with “**×**” in TCGA_PDAC row are not compared as these are PAC mutations data. Among the PDAC samples, red coloured boxes are the mutations which were also observed in TCGA_PDAC (*n* = 185) mutation data. **Fig. 7.** Detection of *KRAS* and *TP53 p.A138V* mutations by allele specific PCR (a-f). **Fig. 8.***KRAS* 12th codon mutation detection by PCR-RFLP method. Lane 1 represent 100 bp DNA ladder. Lane 2 to 11 represents tumour normal paired samples. Mutant samples can be differentiate with presence of 197 fragments in T1, T2, T3, and T5 as *Bst*NI cannot digest mutant containing fragment. Here sample T4-N4 pair is negative control and T5-N5 pair is positive control of NGS cohort. **Fig. 9.***KRAS* mutation screening by different methods in PDAC samples. First row indicates *KRAS* mutation status in 36 PDAC samples. The yellow colour indicates *G12D* mutations, violate colour indicates *G12V* mutation, sky colour indicates *Q61H* mutation, green colour indicates *G12A* mutation, no colour boxes indicate samples with no mutation, and light grey colour indicates samples with failed amplification or poor sequence quality. Other rows indicate different methods (Sanger sequencing, ASPCR, and PCR-RFLP) used for *KRAS* mutation detection. In the methods rows, red colour indicates identification of mutation, white colour indicates mutations could not be identified, and dark ash colour indicates not applicable due to PCR failure for those samples. **Fig. 10.** Identification of p.*A138V* mutation in *TP53* gene by Sanger sequencing. The p.*A138V* mutation in *TP53* identified in 16 patients. Here showing two chromatogram (a&b) for p*.A138V* (Vertical line indicates C/T heterozygous peak) variant identified by Sanger sequencing in 2 patients in the tumour but absent in corresponding normal. **Fig. 11.** Comparison SSE plots between wild type *TP53*and A138Vmut *TP53*. **Fig. 12.** Intermolecular hydrogen bonding analysis between *TP53 (DBD)* and DNA complex with respect to wild type and A138Vmut TP53 protein. Number of intermolecular hydrogen bonds between *TP53*(DBD) (wild type and mutant) and DNA represented in a multivariate plot which shows greater density of data points at 8–12 in wild type (left) and in 5–10 for the mutant (right). Therefore, greater number of intermolecular hydrogen bonds prevailed in the wild type DBD-DNA dynamics simulation than in the corresponding mutant DBD-DNA. **Fig. 13.** Copy number variation plot for *ERBB2* in 93 patients. “X” axis denotes two groups of tissues (Normal and Tumour) of all patients whereas “Y” axis indicates fold change (2^-ΔCT^) of respective groups. **Fig. 14.***KRAS* hotspot region of 8 tumor samples studied by NGS. Showing reads of *KRAS* gene focusing on hotspot codon 12 of 8 samples (**a-h**). Integrative Genomic Browser (IGV) was used for visualization of reads. In figures,“a-h” except “e”, only “C” allele is present in the particular position of *KRAS* gene, whereas figure“e” indicated “C” and mutant “G” alleles in same position. In figure e the variant allele “G” is represented by orange coloured on the reads. **Fig. 15.** Comparison of overall survival of *p*.*A138V* mutant vs. other *TP53* hotspot mutants of TCGA PDAC cohort. Kaplan-Meier survival analysis of all *TP53* hotspot mutants of TCGA PDAC cohortalong with *TP53 A138V* mutants of our patient.

**Additional file 2.** Supplemental methods.

**Additional file 3.**

**Additional file 4.**

**Additional file 5.**

**Additional file 6.**

**Additional file 7.**

**Additional file 8.**

**Additional file 9.**

**Additional file 10.**

**Additional file 11.**

**Additional file 12.**

**Additional file 13.**

## Data Availability

The datasets used and/or analysed during the current study are available from the corresponding author on reasonable request.

## Abbreviations

PDAC: Pancreatic Ductal Adenocarcinoma
PAC: Periampullary Adenocarcinoma
NGS: Next Generation Sequencing
MPS: Massively Parallel Sequencing
TCGA: The Cancer Genome Atlas
COSMIC: Catalogue of Somatic Mutation in Cancer
ASPCR: Allele Specific Polymerase Chain Reaction
AJCC: American Joint Committee on Cancer
WDA: Well Differentiated Adenocarcinoma
MDA: Moderately Differentiated Adenocarcinoma
PDA: Poorly Differentiated Adenocarcinoma
MDS: Molecular Dynamics Simulation
RMSF: Root Mean Square Fluctuation
RMSD: Root Mean Square Deviation
SSE: Secondary Structure Elements
DBD: DNA Binding Domain
CD: Core Domain
OPLS: Optimized Parameters for Liquid Simulation
BSA: Buried Surface Area
CRC: Colorectal Cancer
NSCLC: Non Small Cell Lung Cancer
WES: Whole Exome Sequencing
GDC: Genomic Data Commons
TSG: Tumour Suppressor Gene
IRB: Institutional Review Board
HCC: Hepato Cellular Carcinoma
BPDE: Benzo-(a) pyrenediol-epoxide
RFLP: Restriction Fragment Length Polymorphism
OE: Overexpression
TMA: Tissue Microarray
GOF: Gain Of Function
CA: Complete Absence
CY: Cytoplasmic Staining
MDR: Multifactor Dimensionality Reduction
