# Supplementary material for: A novel hotspot and rare somatic mutation p.A138V, at TP53 is associated with poor survival of pancreatic ductal and periampullary adenocarcinoma patients
Source: Mol Med. 2020 Jun 17;26:59. doi: 10.1186/s10020-020-00183-1 (PMC7302128; doi:10.1186/s10020-020-00183-1)
Supplement: Supplementary file 1 — Additional file 1: Fig. 1. Hematoxilin and Eosin (H&E) staining of types of tumors. a. H&E staining of a PDAC tumor (left 10x magnification, right 20x magnification). b. H&E staining of an intestinal type of PAC tumor (left 10x magnification, right 20x magnification). c. H&E staining of a pancreatobiliary type of PAC tumor (left 10x magnification, right 20x magnification). Fig. 2. Validation of selected variants. Few of the somatic mutations identified in NGS study are being validated by Sanger sequencing method. Validated nucleotides are marked by vertical lines, in each pair upper one is for tumor DNA and lower one is for normal tissue or blood DNA. Fig. 3. Signature of somatic mutations identified in total patient cohort (n = 93). a. In the X axis different types of mutations is given and Y axis denoting frequency of types of mutations. b. Transition and transversion ration of all point mutations. c. In X axis six different signatures of point mutations is given and Y axis denoting the frequency of different signatures. Fig. 4. Frequency of mutations in other recurrently mutated genes in all patients. Different functional domains of proteins are indicated by different colours. The Y axis denotes frequency of mutations and X axis denotes mutations in different positions of the protein domains. Each vertical bar indicates mutation . Recurrent mutations are marked with red circle a.KRAS mutations.b. SMAD4 mutations. c. CTNNB1 mutations. Fig. 5. Damaging and pathogenic variants of non synonymous mutations (n = 57). Damaging and pathogenic variants of non synonymous mutations (n = 57). The dark ass coloured boxes indicates mutations identified as damaging by different functional prediction tools (Provean, SIFT, and Mutation Assessor). The white coloured boxes mean the tolerated mutations whereas light grey coloured boxes mean “unable to identify”. All the dark grey coloured boxes in the ClinVar_Status row indicate pathogenic or likely pathogenic variants and black col [file 10020_2020_183_MOESM1_ESM.docx]

**Supplemental Figures**

**a**

**
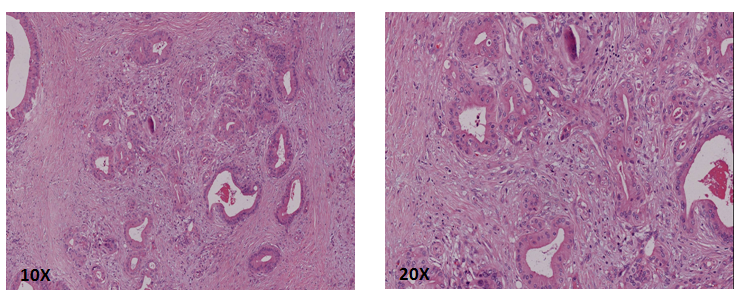
**

**b**

**
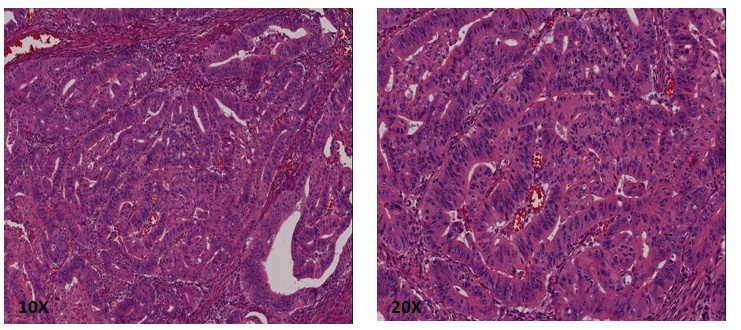
**

**c**

**
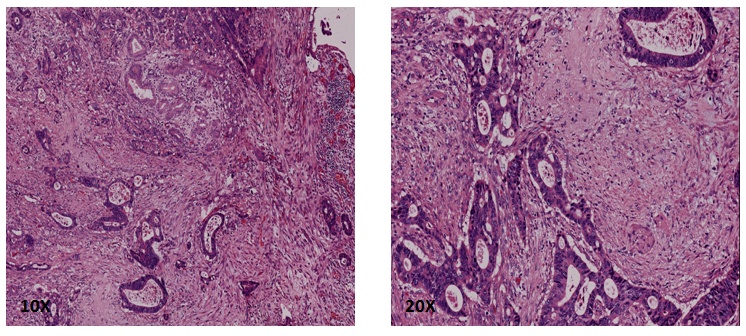
**

**S-Fig.1. Hematoxilin and Eosin (H&E) staining of types of tumors.** **a**. H&E staining of a PDAC tumor (left 10x magnification, right 20x magnification). **b**. H&E staining of an intestinal type of PAC tumor (left 10x magnification, right 20x magnification). **c**. H&E staining of a pancreatobiliary type of PAC tumor (left 10x magnification, right 20x magnification).

**a**

**
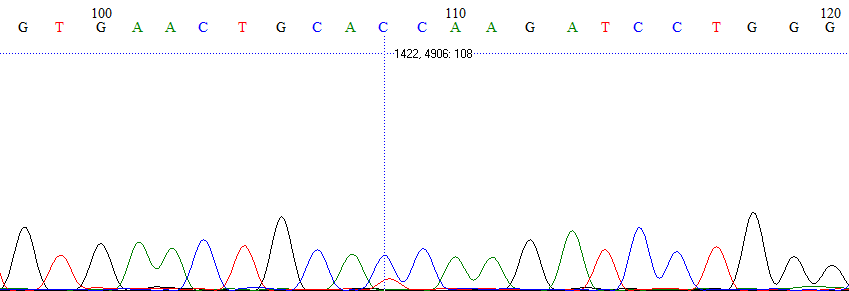
**

**
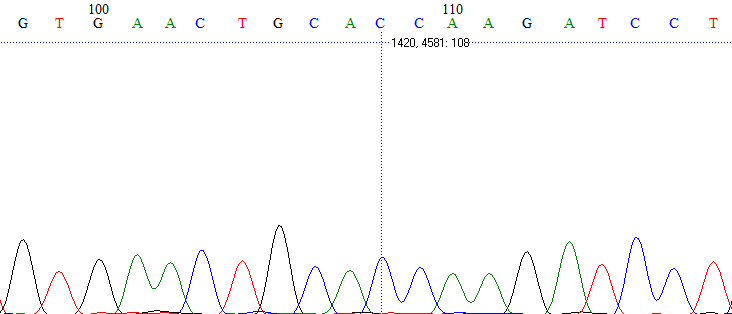
**

**b**

**
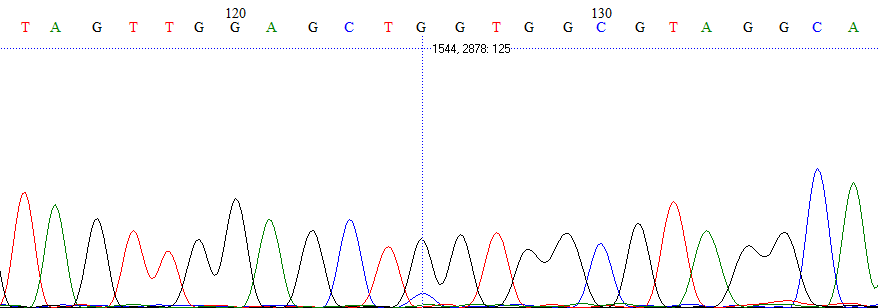
**

**
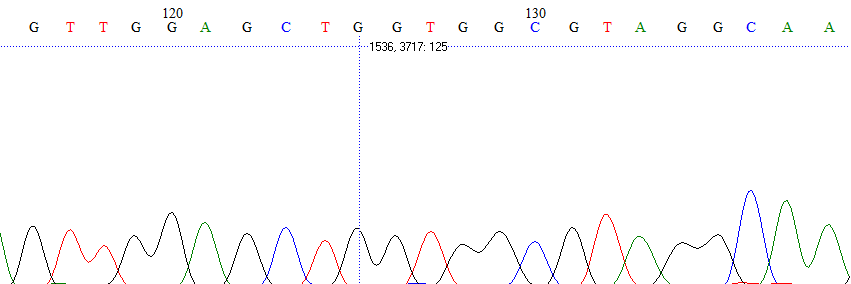
**

**c**

**
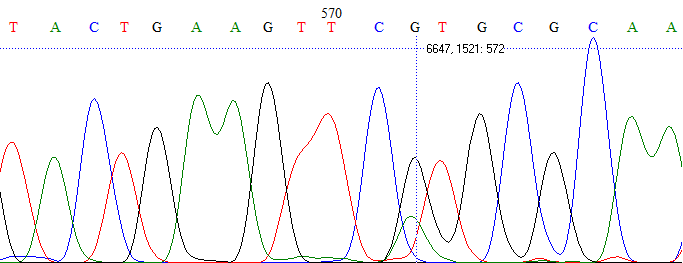
**

**
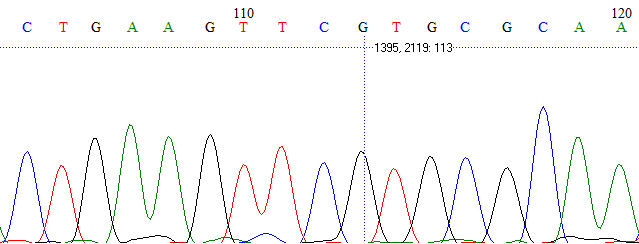
**

**d**


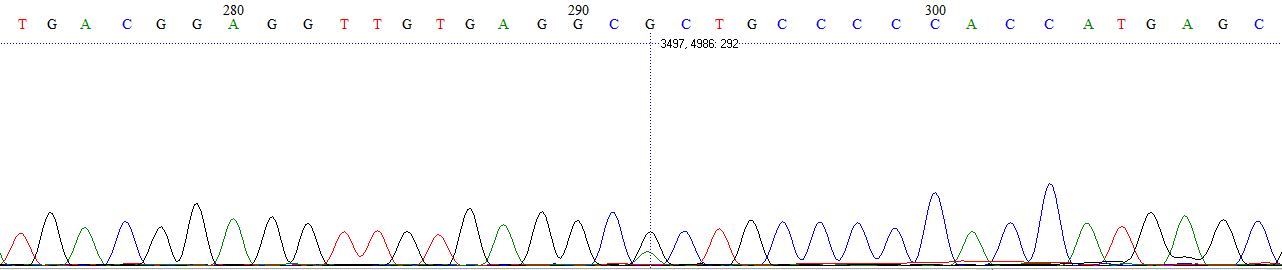


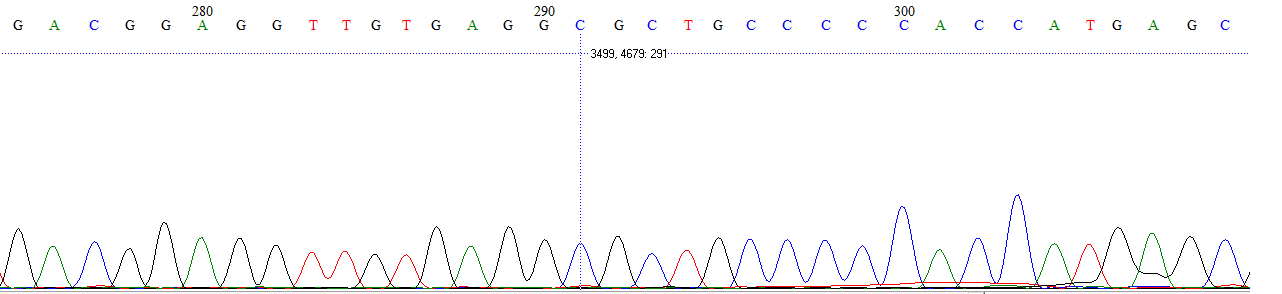


**e**


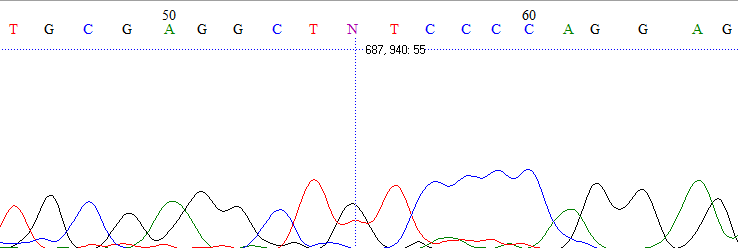


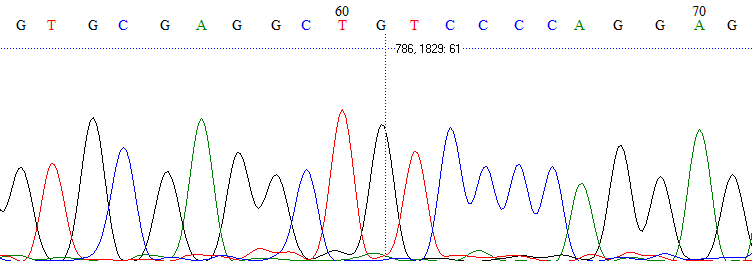


**f**


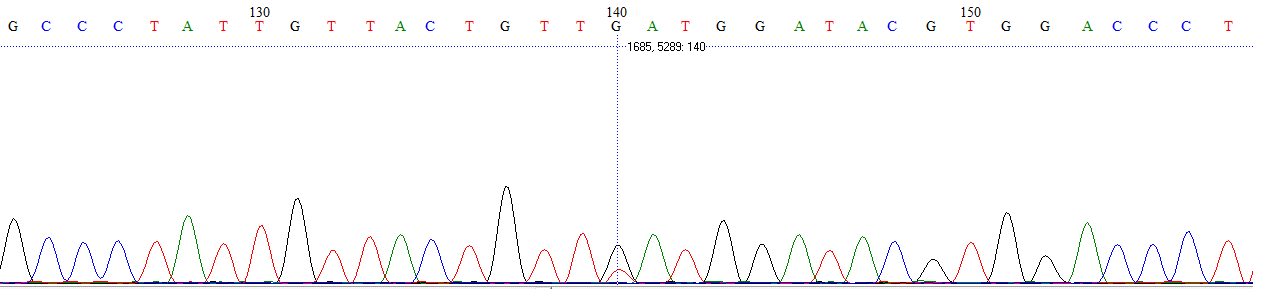


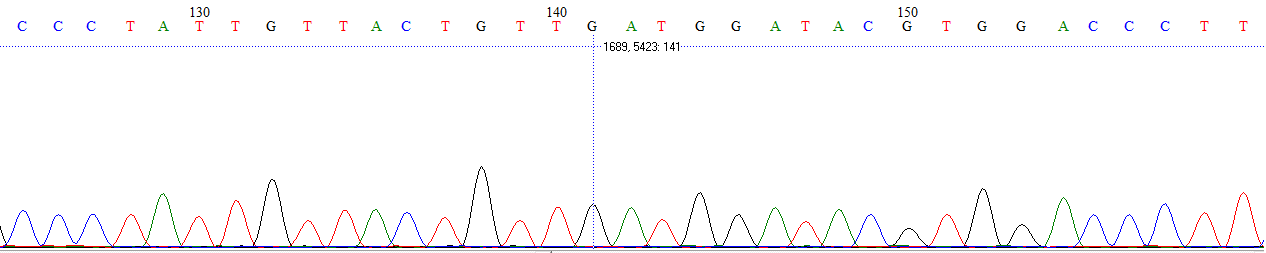


**g**


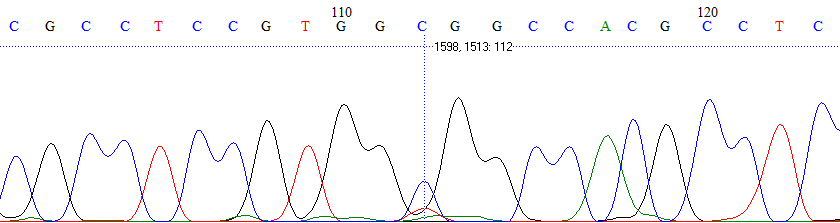


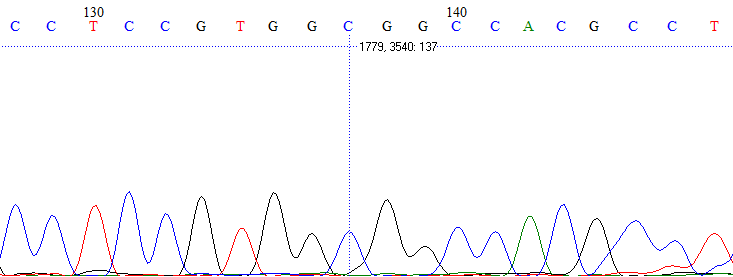


**h**


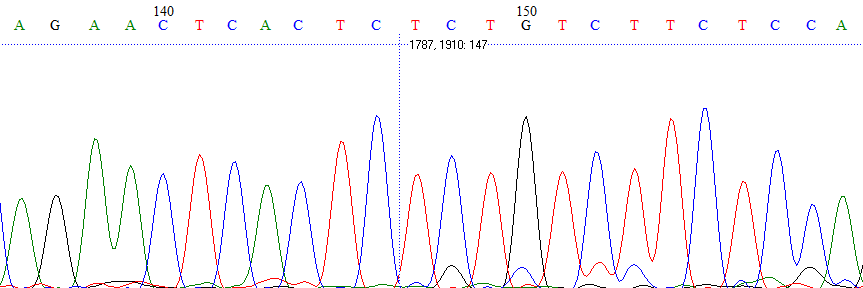


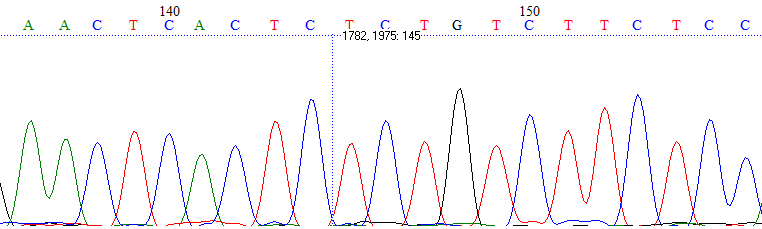


**i**


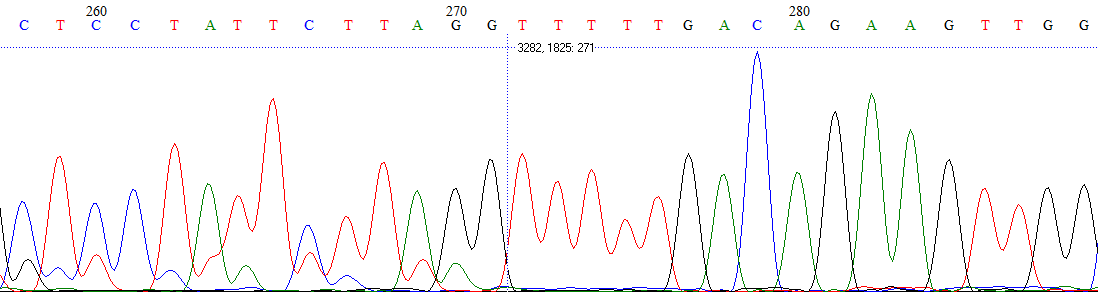


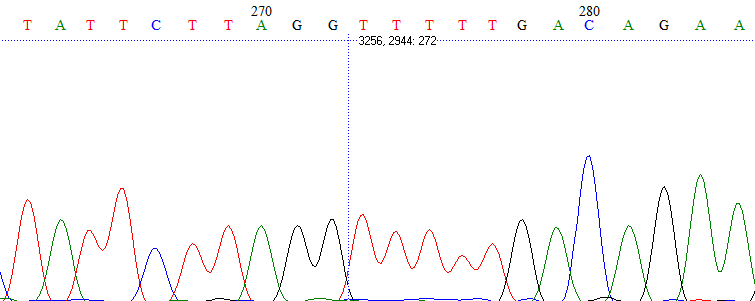


**S-Fig.2. Validation of selected variants.** Few of the somatic mutations identified in NGS study are being validated by Sanger sequencing method. Validated nucleotides are marked by vertical lines, in each pair upper one is for tumor DNA and lower one is for normal tissue or blood DNA.

**a**. Chromatogram of tumour-normal pair tissues for *ERBB3*:p.*T355I*. **b**. Chromatogram oftumour-normal pair tissues for*KRAS*:p.*G12R.***c**. Chromatogram oftumour normal pair for*ERCC1*:p.*V116M*.**d**. Chromatogram of tumournormal pair tissues for*TP53:*p.R43H. **e**. Chromatogram oftumour -normal pair tissues for*ITGB3*:p.*C432F*. **f**. Chromatogram oftumour- normal pair tissues for*SMAD4*:p.*D351Y*.**g**. Chromatogram oftumour- normal pair tissues for*PARP1:*p*.A366V.* **h**. Chromatogram oftumour- normal pair tissues for *AGAP2*:p*.S439fs.* **i**. Chromatogram oftumour- normal pair tissues for *BIRC6*:p.*R3688fs*


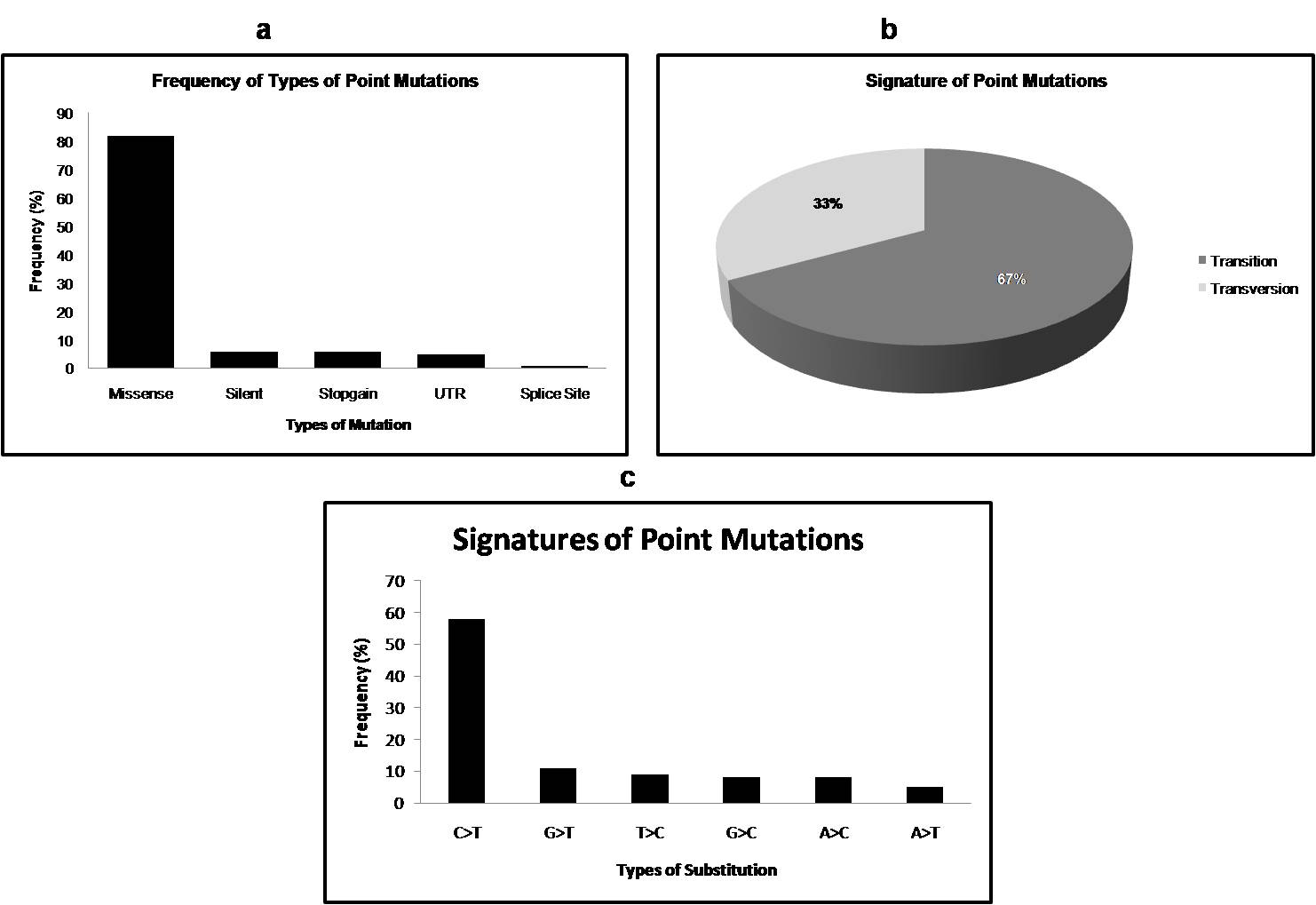


**S-Fig.3. Signature of somatic mutations identified in total patient cohort (n=93).** **a.** In the X axis different types of mutations is given and Y axis denoting frequency of types of mutations. **b.** Transition and transversion ration of all point mutations. **c.** In X axissix different signatures of point mutations is given and Y axis denoting the frequency of different signatures.


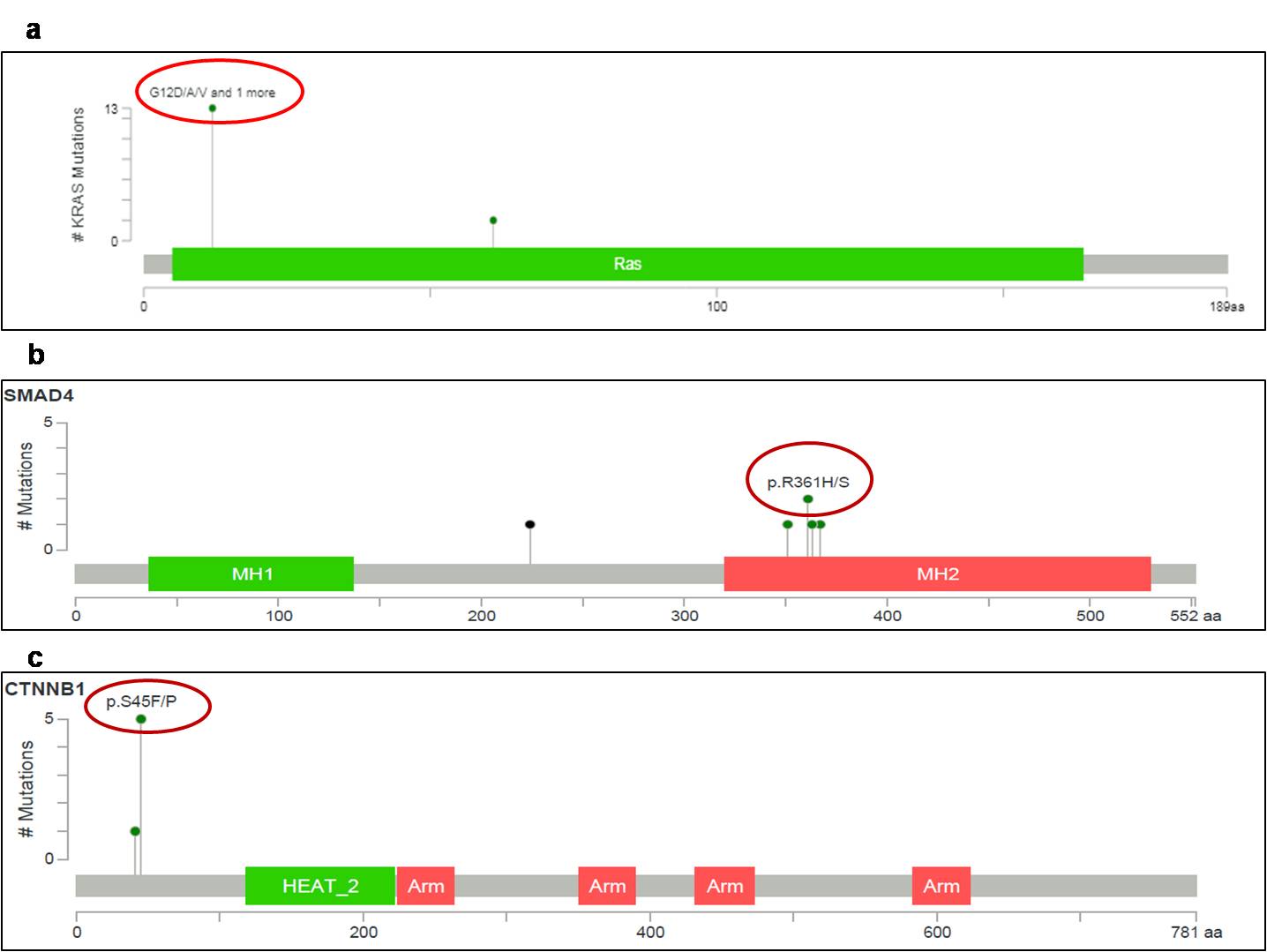


**S-Fig.4.** **Frequency of mutations in other recurrently mutated genes in all patients.** Different functional domains of proteins are indicated by different colours. The Y axis denotes frequency of mutations and X axis denotes mutations in different positions of the protein domains. Each vertical bar indicates mutation . Recurrent mutations are marked with red circle **a.** *KRAS* mutations.**b**. *SMAD4* mutations. **c**. *CTNNB1* mutations.


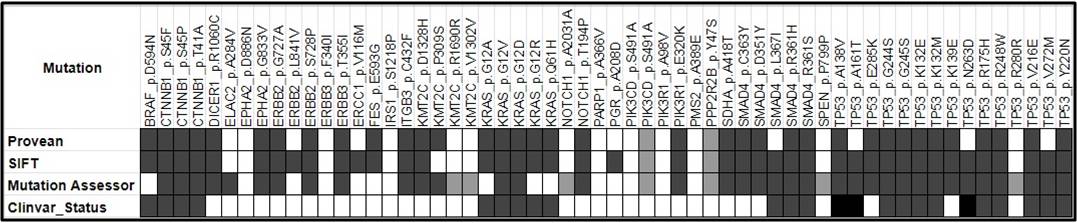


**S-Fig.5. Damaging and pathogenic variants of non synonymous mutations (n=57).** Damaging and pathogenic variants of non synonymous mutations (n=57). The dark ass coloured boxes indicates mutations identified as damaging by different functional prediction tools *(Provean, SIFT, and Mutation Assessor)*. The white coloured boxes mean the tolerated mutations whereas light grey coloured boxes mean “unable to identify”. All the dark grey coloured boxes in the *ClinVar_Status* row indicate pathogenic or likely pathogenic variants and black coloured boxes are reported as uncertain significance in *ClinVar* database.

**
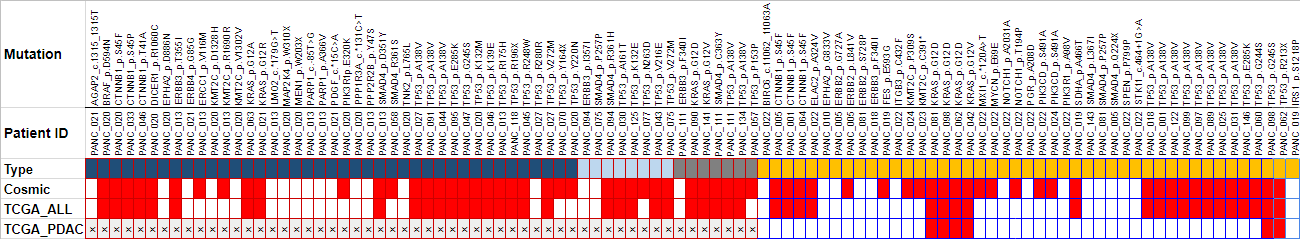
**

**S-Fig.6.Comparison of reported and novel variants observed in this study with those reported in TCGA and Cosmic database.**These are the total mutations identified by both NGS and Sanger sequencing method. The blue coloured boxes in sample row are PAC mixed samples, Sky coloured boxes are PAC intestinal samples, Grey coloured boxes are PAC pancreatobiliary samples and yellow coloured boxes are PDAC samples. The red colouredboxes in COSMIC and TCGA row are the reported mutation in these databases. The boxes marked with “**×**” in TCGA_PDAC row are not compared as these are PAC mutations data. Among the PDAC samples, red coloured boxes are the mutations which were also observed in TCGA_PDAC (n=185) mutation data.

**a**


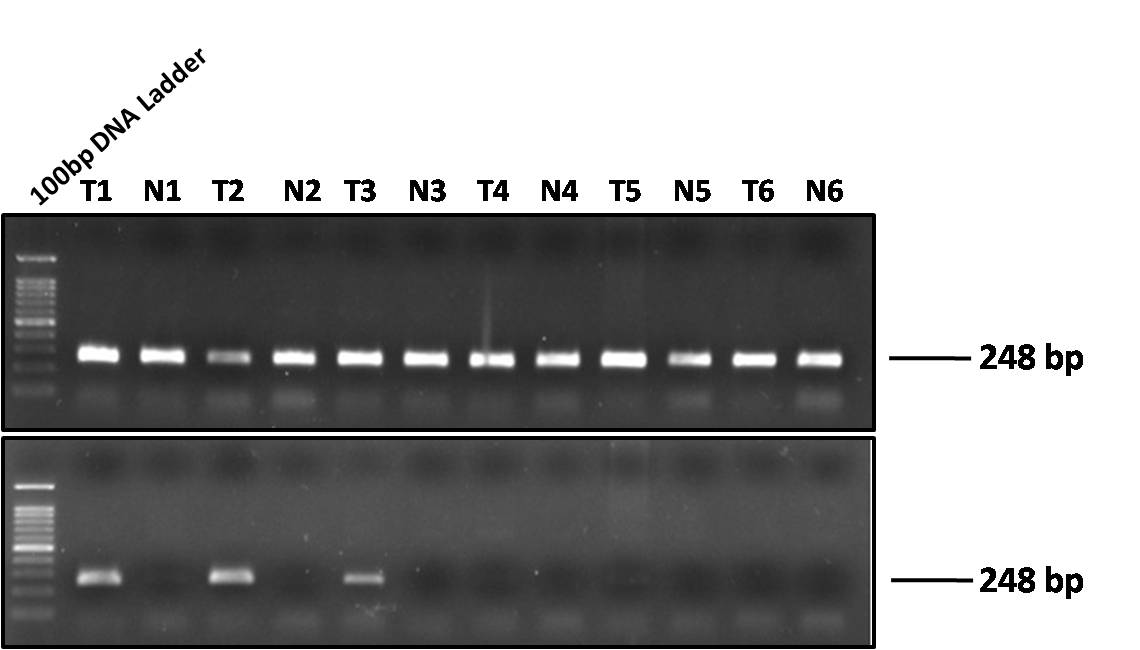


**b**
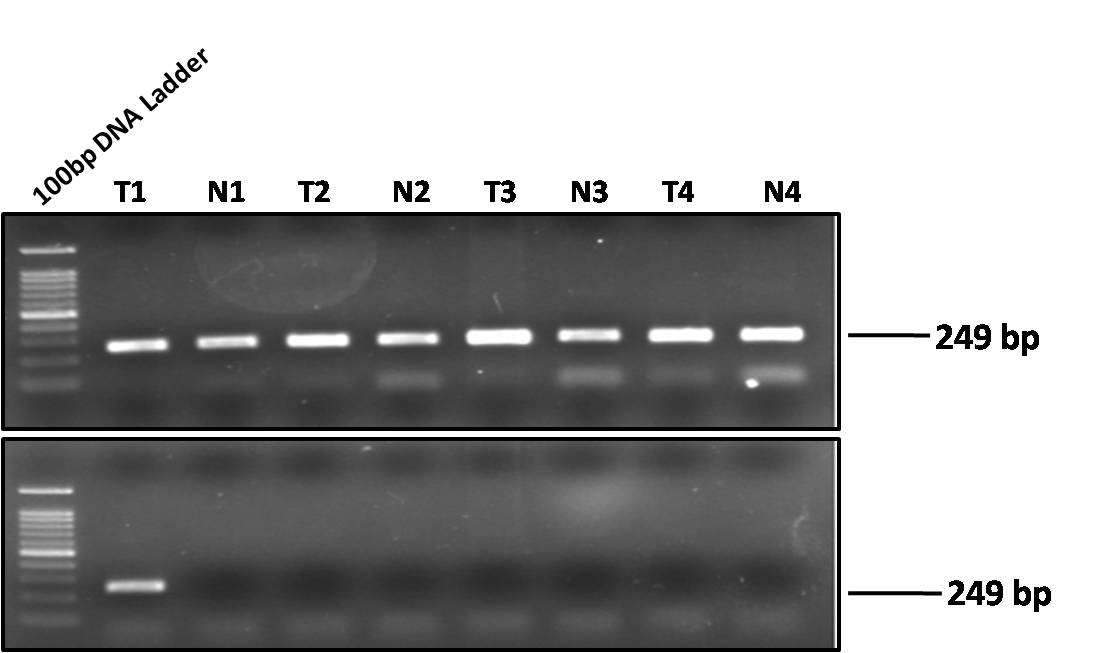


**c**


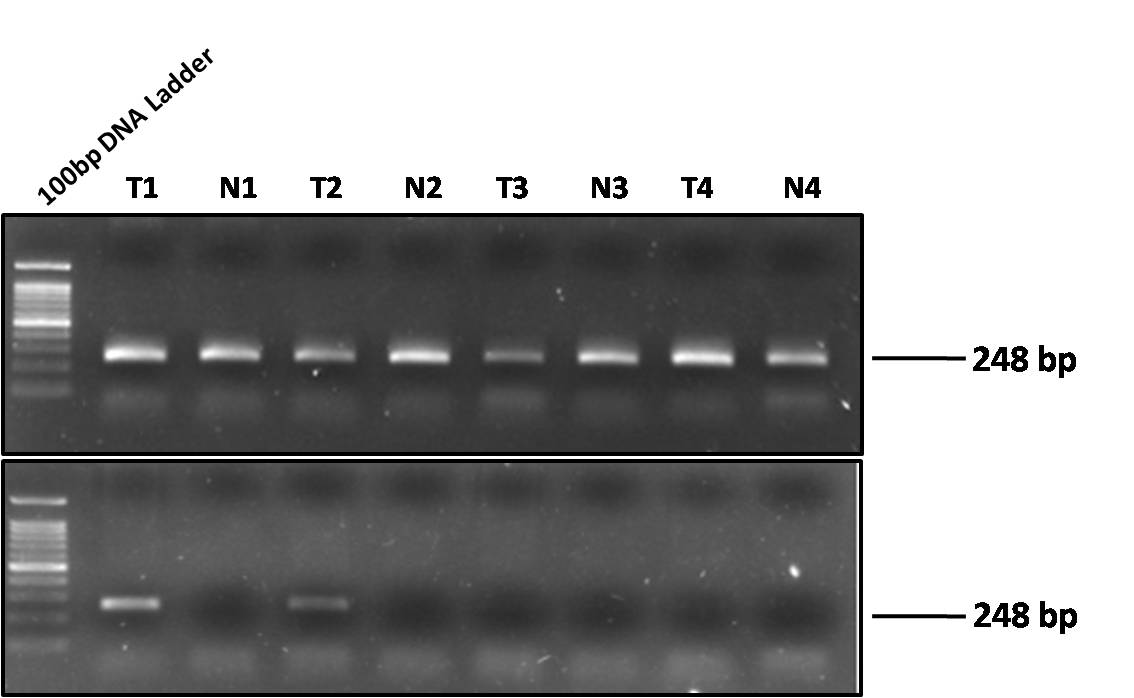


**d**


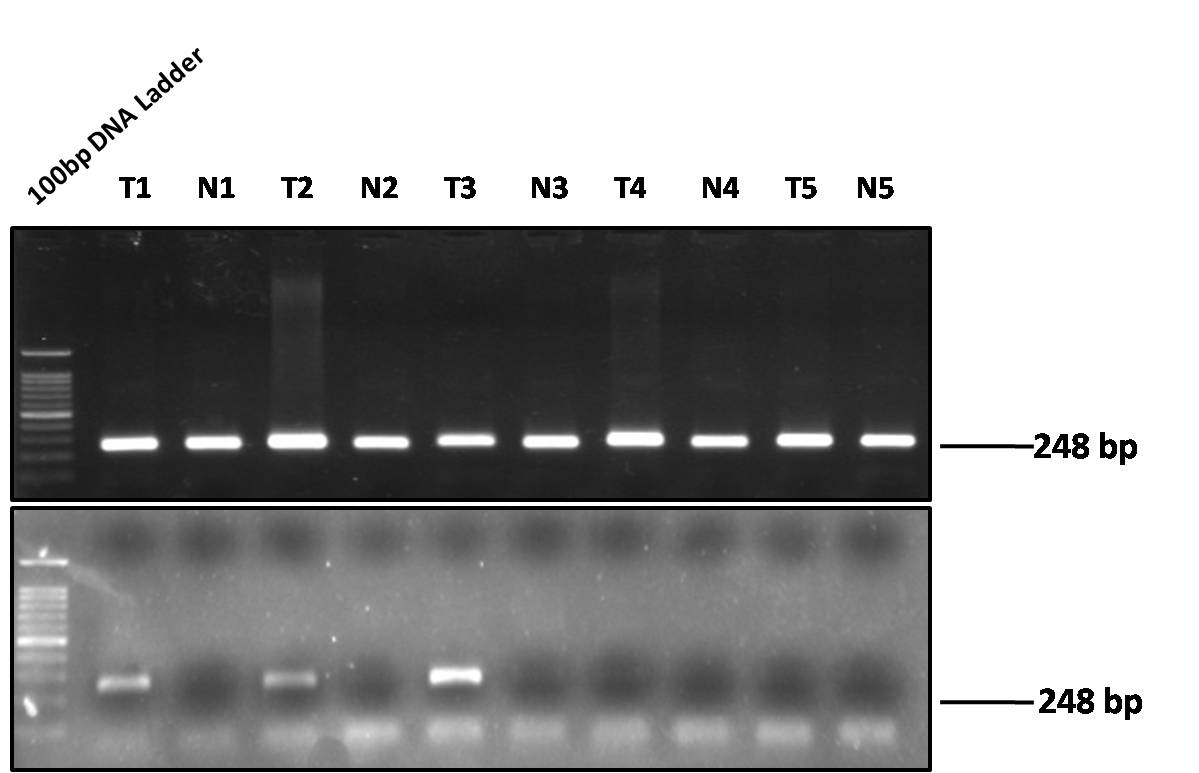


**e**


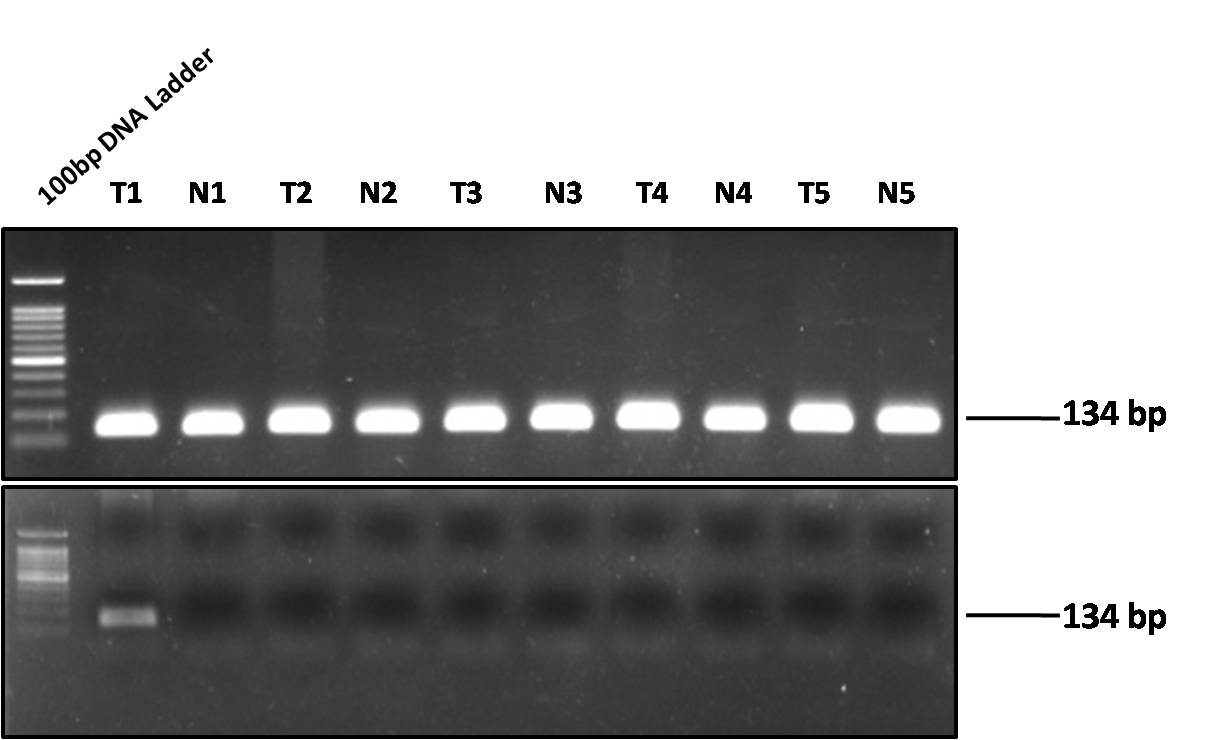


**f**


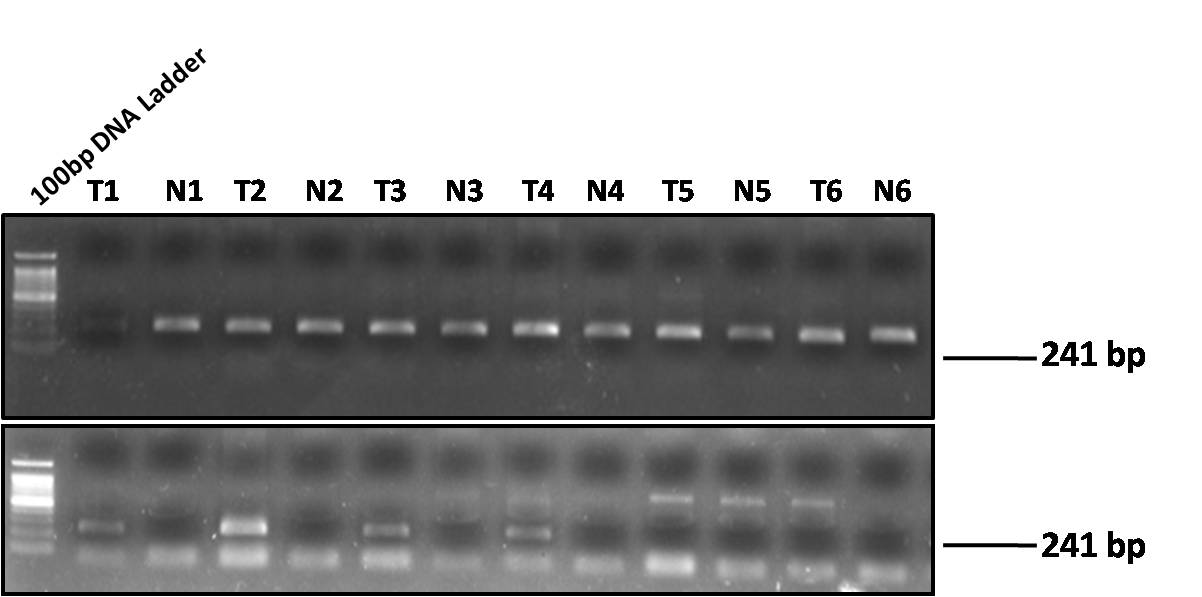


**S-Fig.7.Detection of *KRAS* and *TP53 p.A138V* mutations by allele specific PCR (a-f)**.

**a.**Detection of *KRAS G12A* mutation: Lane 2 to 13 represents 6 tumour-normal paired samples. In the upper panel PCR was done with primer correspond to wild type allele. A single band observed in all the samples due to presence of normal allele. In the lower panel PCR was done with allele specific mutant primer . A single strong band was observed in 3 tumour sample (T1, T2, and T3) but not in the corresponding normal sample (N1, N2, N3). Other 3 pairs of samples were used as negative control for allele specific mutant primer.

**b.**Detection of *KRAS G12R* mutation by ASPCR: Lane 2 to 9 represents 4 tumour-normal paired samples. In the upper panel PCR was done with primer correspond to wild type allele. A single band observed in all the samples due to presence of normal allele. In the lower panel PCR done with allele specific mutant primer. A single strong band observed in 1 tumour sample (T1) but not in the corresponding normal sample (N1). Other 3 pairs of samples were used as negative control from allele specific mutant primer.

**c.**Detection of *KRAS G12V* mutation Lane 2 to 9 represents 4 tumour normal- paired samples. In the upper panel PCR was done with primer correspond to wild type allele. A single band observed in all the samples due to presence of normal allele. In the lower panel PCR done with allele specific mutant primer. A single strong band observed in 2 tumour samples (T1, and T2) but not in the corresponding normal sample (N1, and N2). Other 2 pairs of samples were used as negative control for allele specific mutant primer.

**d.**Detection of *KRAS G12D* mutation: Lane 2 to 11 represents 5tumour- normal paired samples. In the upper panel PCR was done with primer correspond to wild type allele. A single band observed in all the samples due to presence of normal allele. In the lower panel PCR was done with allele specific mutant primer. A single strong band observed in 3 tumour sample (T1, T2, and T3) but not in the corresponding normal sample (N1, N2, and N3). Other 2 pairs of samples were used as negative control for allele specific mutant primer.

**e.**Detection of *KRAS Q61H* mutation: Lane 2 to 11 represents 5 tumour normal -paired samples. In the upper panel PCR was done with primer correspond to wild type allele. A single band observed in all the samples due to presence of normal allele. In the lower panel PCRwas done with allele specific mutant primer. A single strong band observed in 1tumour sample (T1) but not in the corresponding normal sample (N1).Other 4 pairs of samples were used as negative control for allele specific mutant primer.

**f.**Detection of *TP53 p.A138V* mutation: Lane 2 to 13 represents 6tumour- normal paired samples. In the upper panel PCR was done with primer correspond to wild type allele. A single band observed in all the samples due to presence of normal allele. In the lower panel PCR done with allele specific mutant primer. A single strong band observed in 4 tumour sample (T1, T2, T3, & T4) but not in the corresponding normal sample (N1, N2, N3, & N4). Other 4 pairs of samples were used as negative control for allele specific mutant primer.
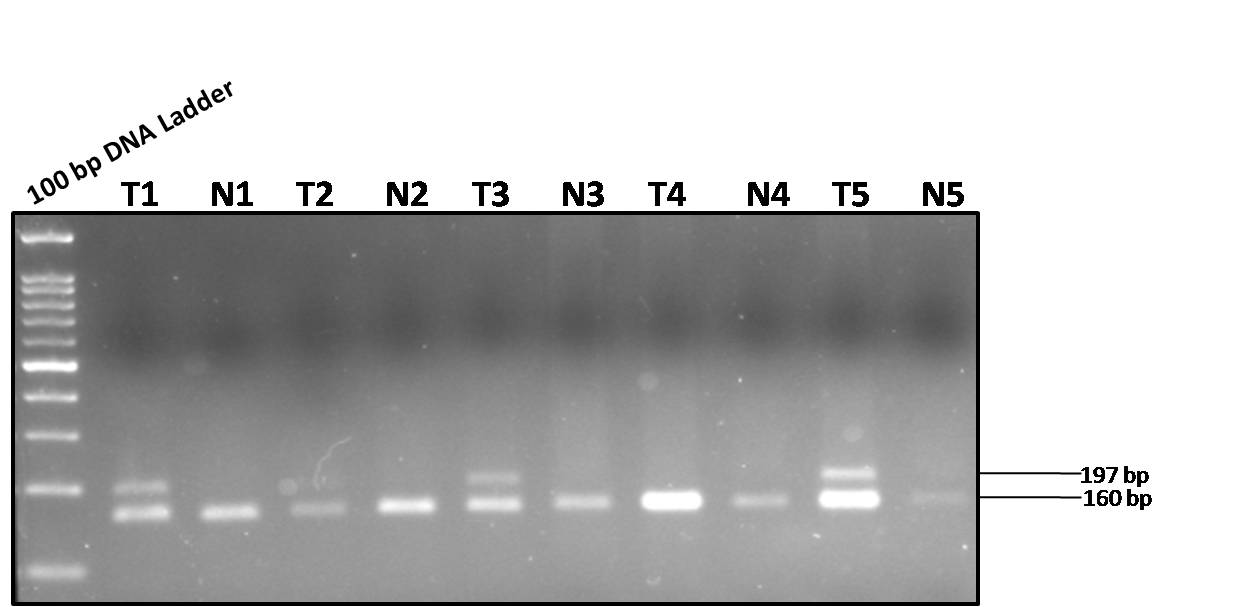


**S-Fig.8. *KRAS* 12^th^ codon mutation detection by PCR-RFLP method.** Lane 1 represent 100bp DNA ladder. Lane 2 to 11 represents tumour normal paired samples. Mutant samples can be differentiate with presence of 197 fragments in T1, T2, T3, and T5 as *Bst*NI cannot digest mutant containing fragment. Here sample T4-N4 pair is negative control and T5-N5 pair is positive control of NGS cohort.


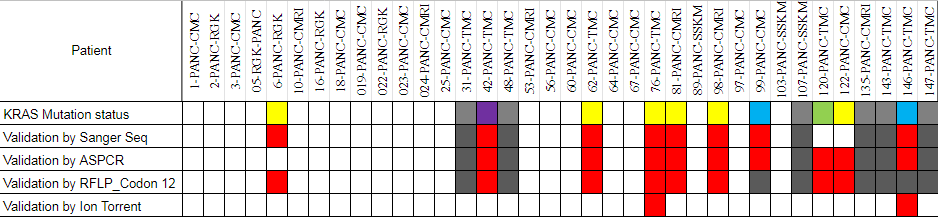


**S-Fig.9**. ***KRAS* mutation screening by different methods in PDAC samples.** First row indicates *KRAS* mutation status in 36 PDAC samples. The yellow colour indicates *G12D* mutations, violate colour indicates *G12V* mutation, sky colour indicates *Q61H* mutation, green colour indicates *G12A* mutation, no colour boxes indicate samples with no mutation, and light grey colour indicates samples with failed amplification or poor sequence quality. Other rows indicate different methods (Sanger sequencing, ASPCR, and PCR-RFLP) used for *KRAS* mutation detection. In the methods rows, red colour indicates identification of mutation, white colour indicates mutations could not be identified, and dark ash colour indicates not applicable due to PCR failure for those samples.

**a**

**Tumour**


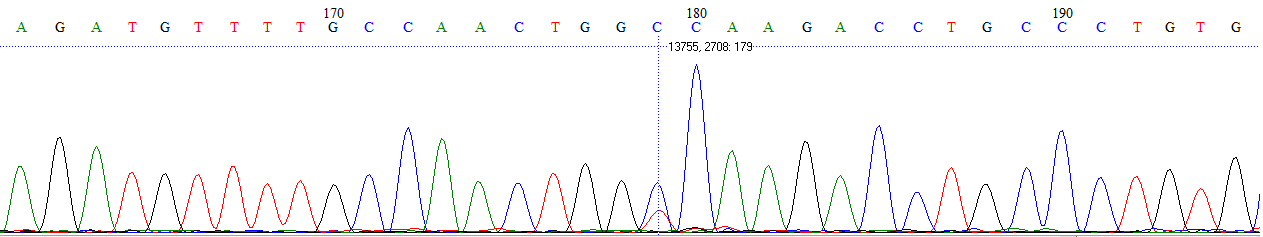


**Normal**


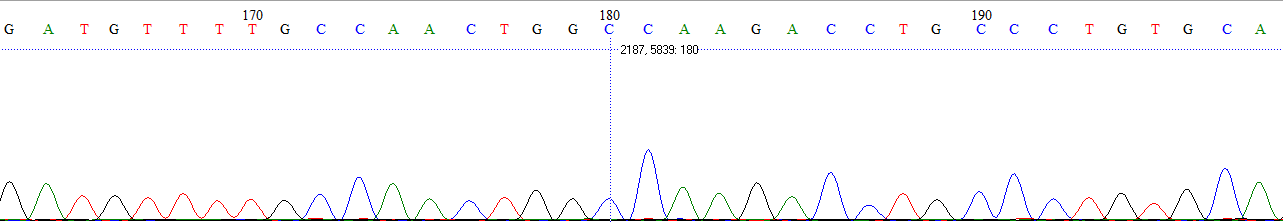


**b**

**Tumour**


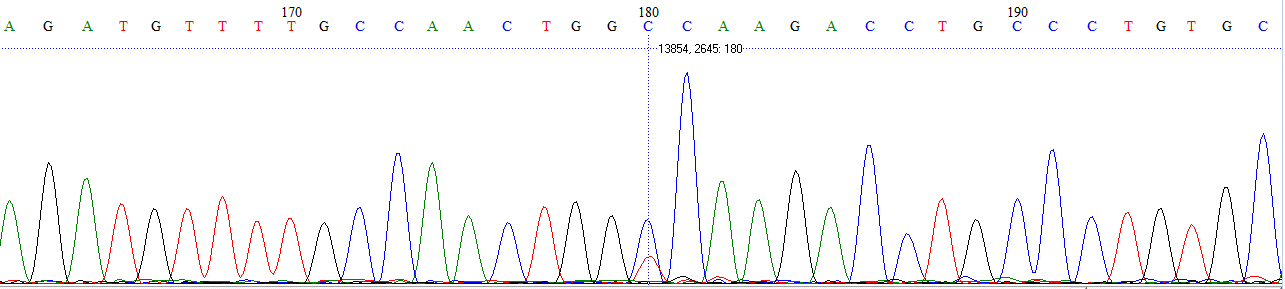


**Normal**


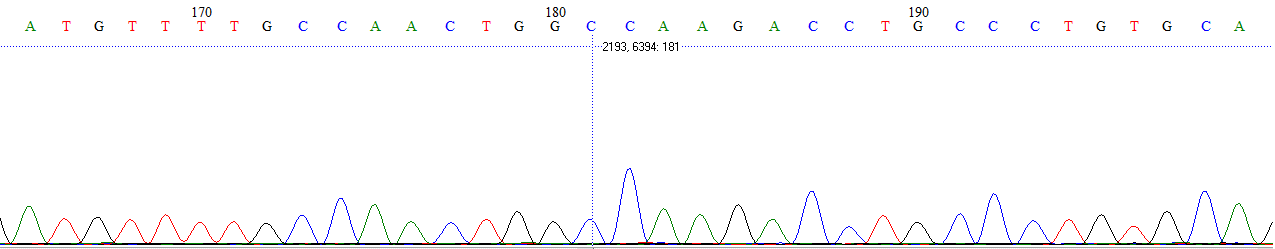


**S-Fig.10.** **Identification of p.*A138V* mutation in *TP53* gene by Sanger sequencing.** The p.*A138V* mutation in *TP53* identified in 16 patients. Here showing two chromatogram (a&b) for p*.A138V* (Vertical line indicates C/T heterozygous peak) variant identified by Sanger sequencing in 2 patients in the tumour but absent in corresponding normal.


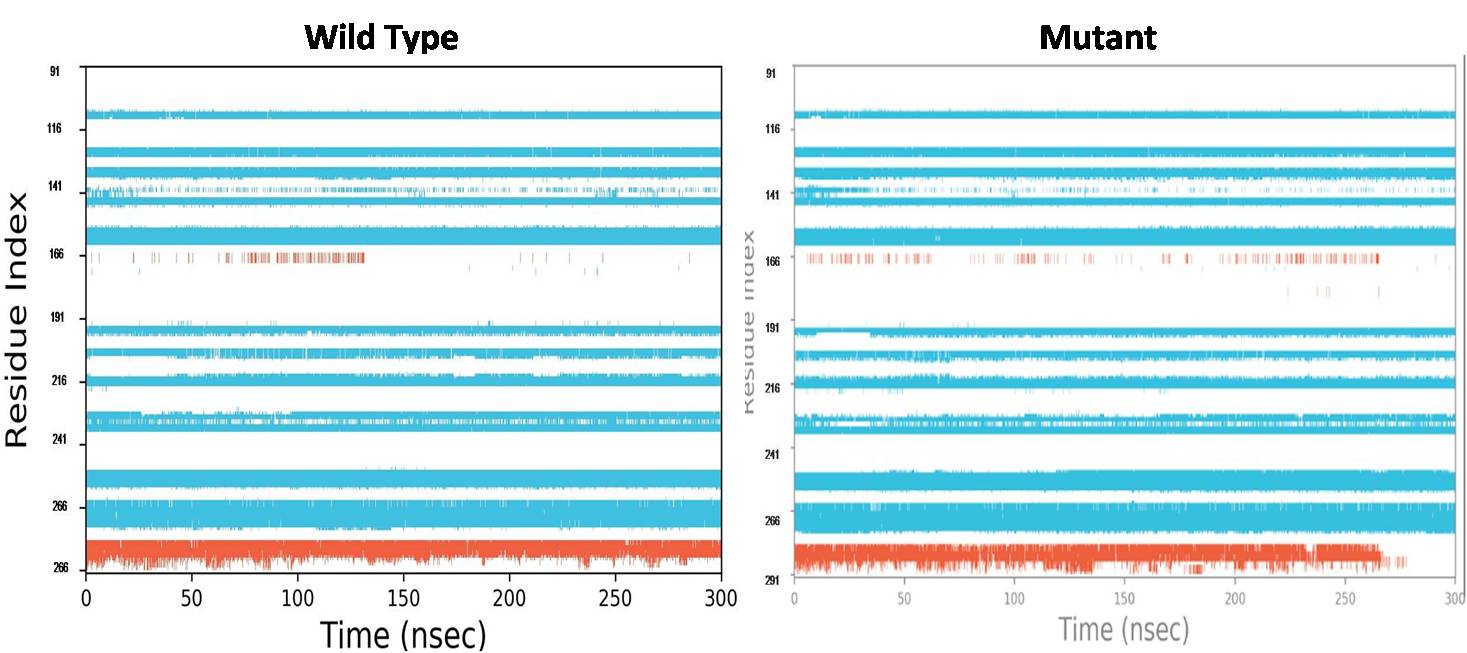


**S-Fig.11. Comparison SSE plots between wild type *TP53*and A138Vmut *TP53*.**

The plot represents time dependent SSE of the *TP53* DNA binding domain of wild type and p.*A138V* mutant respectively. The turquoise blue colour indicatedbeta-strands; Orange red colour indicatedalpha-helices and White space indicate loops and turns.


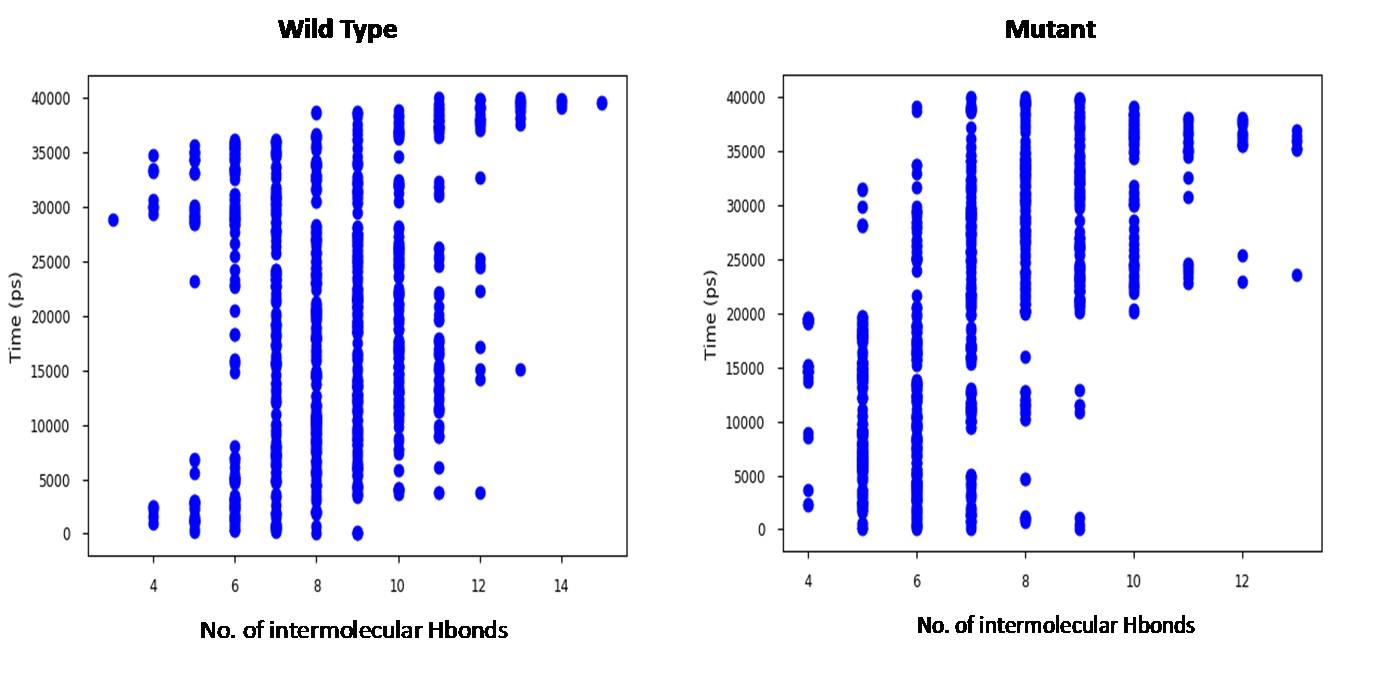


**S-Fig.12. Intermolecular hydrogen bonding analysis between *TP53 (DBD)* and DNA complex with respect to wild type and A138Vmut TP53 protein.** Number of intermolecular hydrogen bonds between *TP53*(DBD) (wild type and mutant) and DNA represented in a multivariate plot which shows greater density of data points at 8-12 in wild type (left) and in 5-10 for the mutant (right). Therefore, greater number of intermolecular hydrogen bonds prevailed in the wild type DBD-DNA dynamics simulation than in the corresponding mutant DBD-DNA.


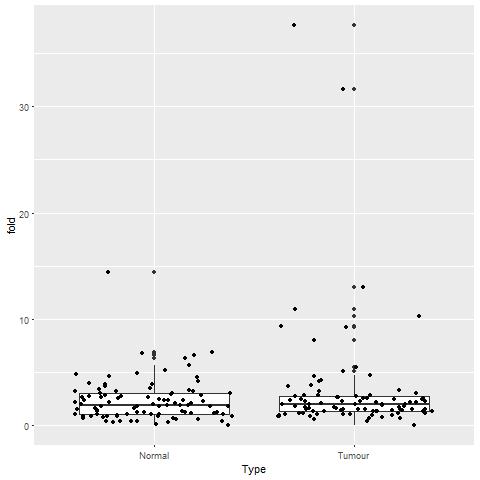


**S-Fig.13.Copy number variation plot for *ERBB2* in 93 patients.** “X” axis denotes two groups of tissues (Normal and Tumour) of all patients whereas “Y” axis indicates fold change (2^-ΔCT^) of respective groups.

**a**


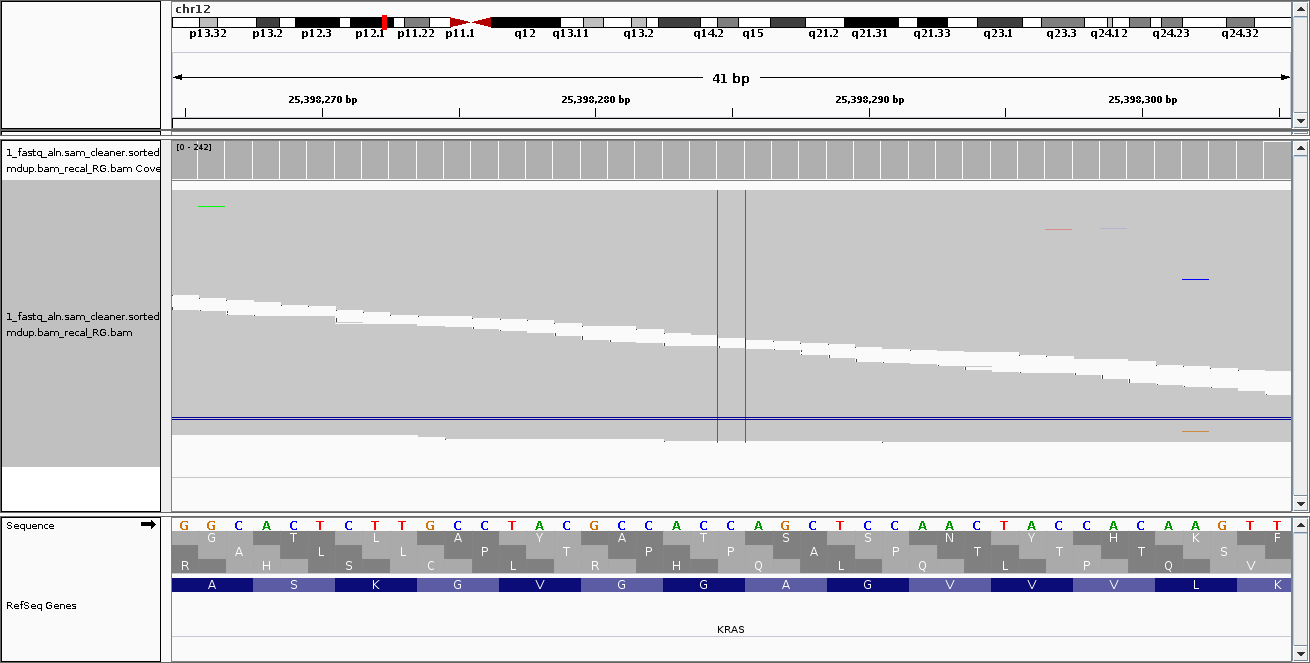


**b**


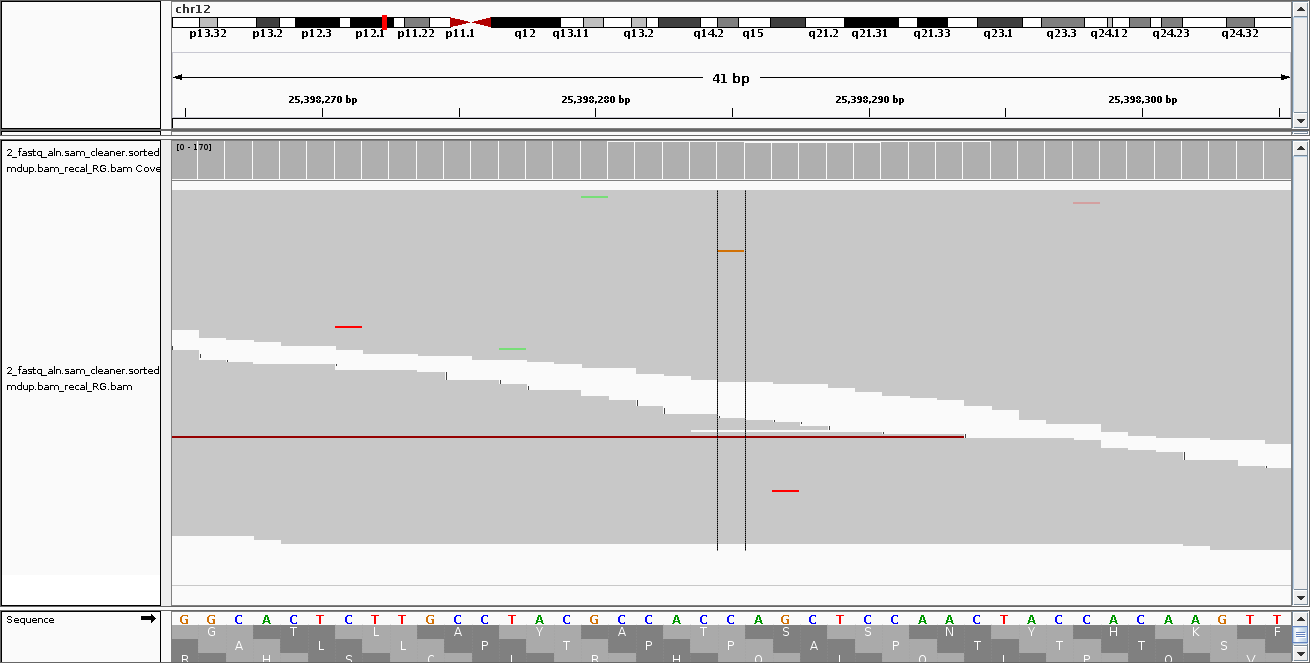


**c**


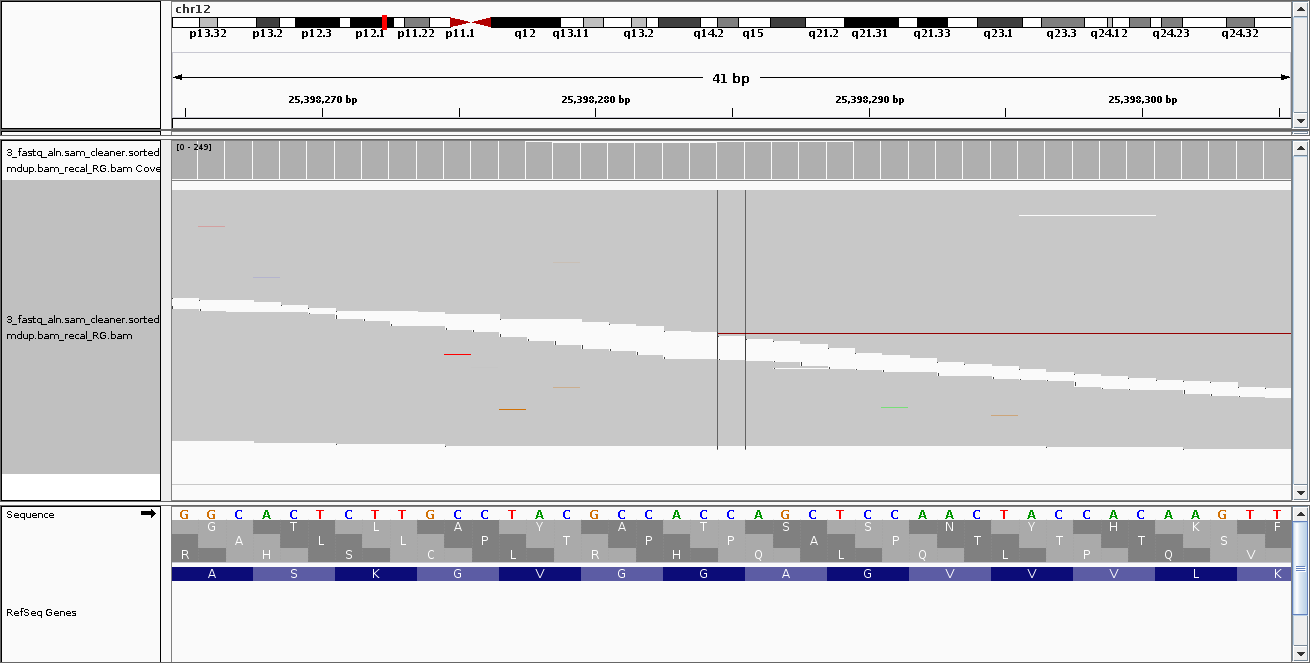


**d**


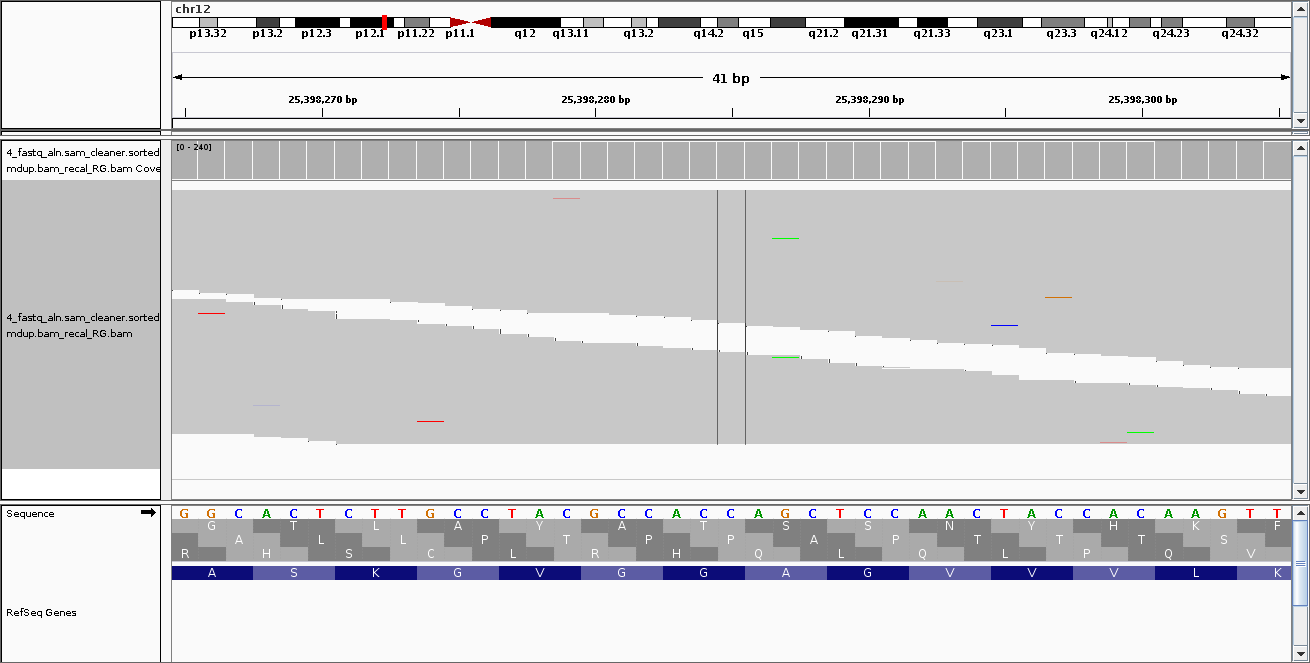


**e**


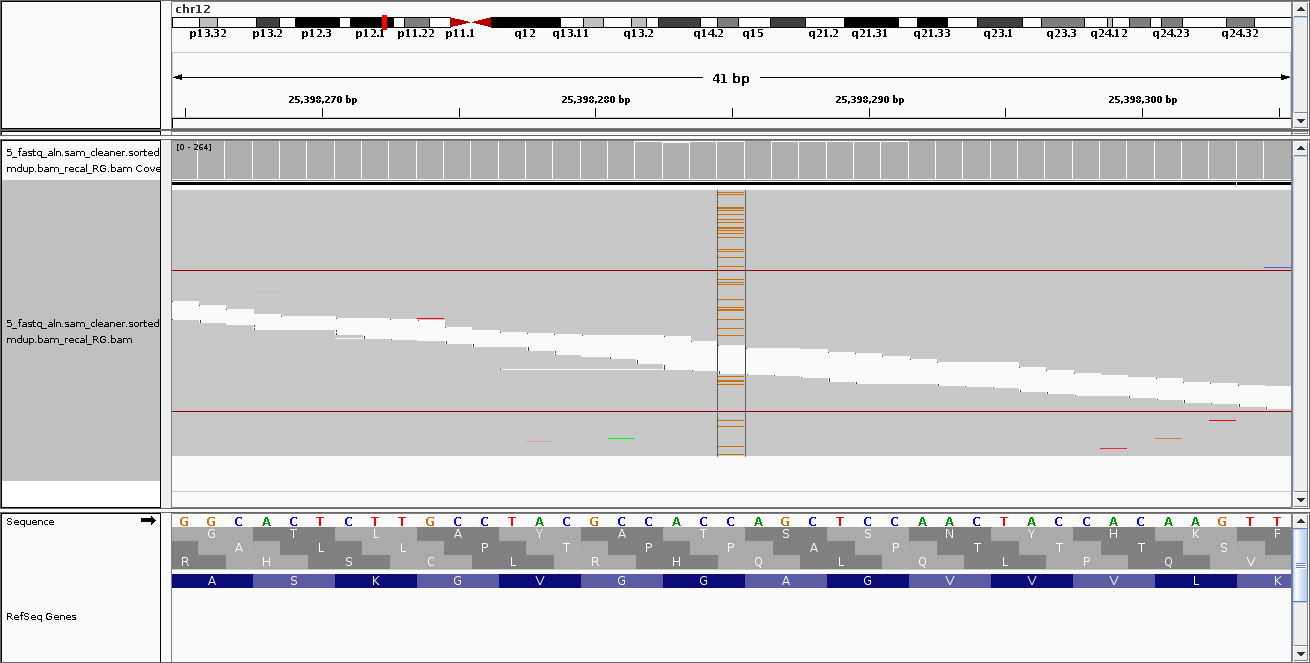


**f**


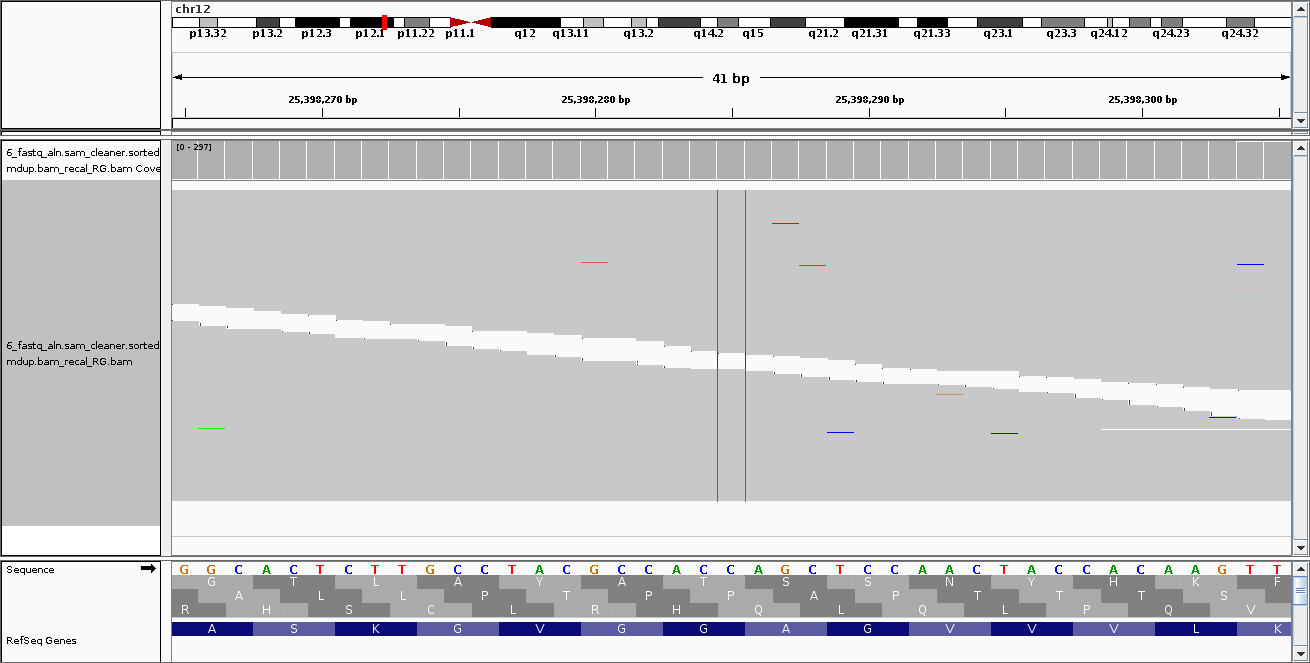


**g**


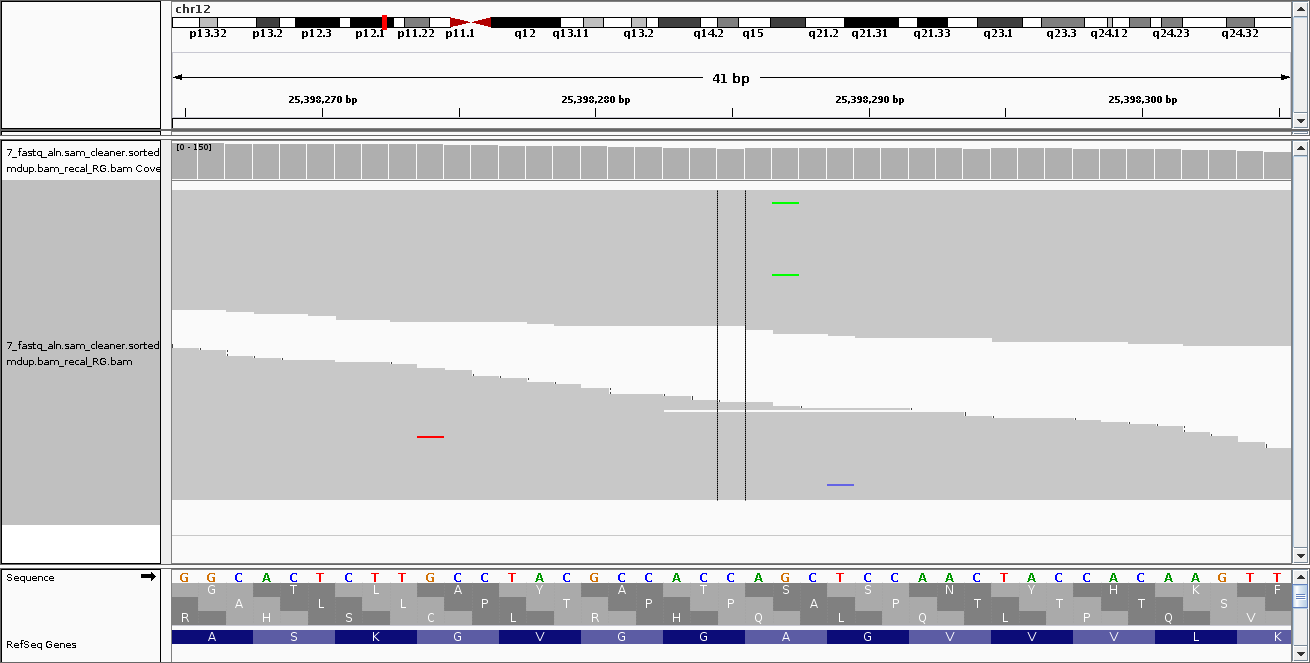


**h**


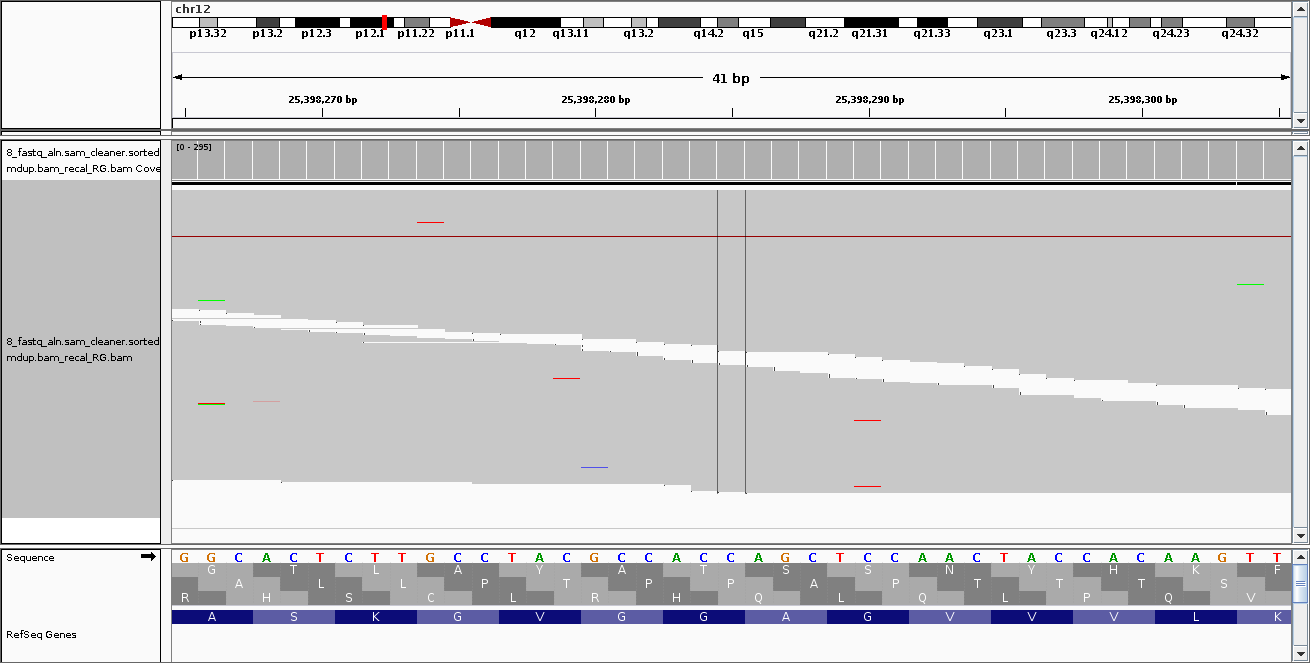


**S-Fig.14: *KRAS* hotspot region of 8 tumor samples studied by NGS .**Showing reads of *KRAS* gene focusing on hotspot codon 12 of 8 samples (**a-h**). Integrative Genomic Browser (IGV) was used for visualization of reads. In figures,“a-h” except “e”, only “C” allele is present in the particular position of *KRAS* gene, whereas figure“e” indicated “C” and mutant “G” alleles in same position. In figure e the variant allele “G” is represented by orange coloured on the reads


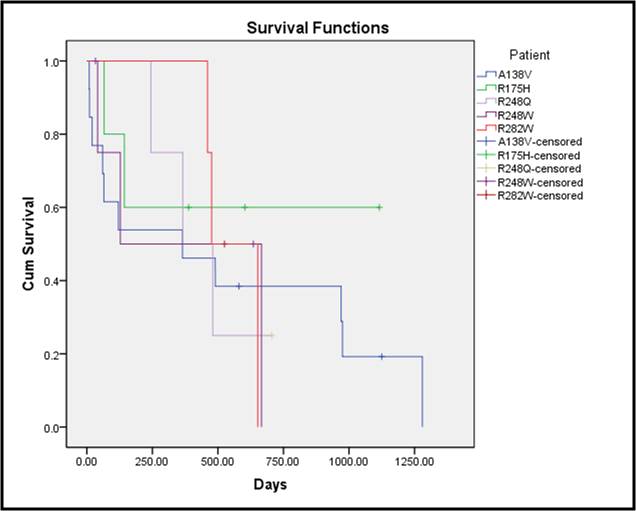


**S-Fig.15**. **Comparison of overall survival of p.*A138V* mutant vs. other *TP53* hotspot mutants of TCGA PDAC cohort.** Kaplan-Meier survival analysis of all *TP53* hotspot mutants of TCGA PDAC cohortalong with *TP53A138V* mutants of our patient.
