# Supplementary material for: A novel hotspot and rare somatic mutation p.A138V, at TP53 is associated with poor survival of pancreatic ductal and periampullary adenocarcinoma patients
Source: Mol Med. 2020 Jun 17;26:59. doi: 10.1186/s10020-020-00183-1 (PMC7302128; doi:10.1186/s10020-020-00183-1)
Supplement: Supplementary file 2 — Additional file 2. Supplemental methods. [file 10020_2020_183_MOESM2_ESM.docx]

**Supplemental Information**

***ERBB2/Her2-Neu* amplification detection by TaqMan assay**

PCR was done in a total volume of 5 ul consisting of 2 ul of genomic DNA (10ng/ul), 2.5 ul of 2x TaqMan Genotyping Master Mix (Rosche, Germeny), 0.25ul of TaqMan probe (20X, FAM dye) and 0.25 ul of H_2_0. Quantitative PCR was performed on an ABI 7900HT Real Time PCR system (Applied Biosystems, CA, USA). The PCR condition as follows: 10 min in 95°C followed by 40 cycles of denaturation for 15 seconds at 95°C, and annealing for 1 min at 60°C. *RNaseP* (Applied Biosciences, CA, USA) (20X, VIC dye) used as a reference control with 2 copies in the human genome (not needed so much details). Target and reference assays that were used for copy number calculation, were derived from the mean of duplicate Ct values.

**Genotyping of key polymorphisms of TP53 and its associated genes**

The Arg72Pro polymorphism (rs1042522) of *TP53* was genotyped by PCR-RFLP method. The PCR product was amplified with respective primers listed in supplemental Table 2. Annealing temperature for PCR was standardized at 60°C for 45 sec. The *BstU*I enzyme (5U)  (New England Biolabs, Beverey, Massachusetts, United States) at 60°C was used to determine the genotype, which cuts the PCR product of 279 bp in presence of ‘*G*’ allele (Pro), generated 160, and 119 bp fragments. Whereas, in presence of ‘*C*’ allele (Arg), the enzyme did not cut the PCR product. The genotype of “CC” was detected by 279 bp band size. The heterozygous genotype “GC” was detected by, presence of 279bp, 160bp, and 119bp band sizes.

*TP53 PIN3 Ins16bp*polymorphism (rs17878362) was detected by amplifying genomic DNA with respective primers (listed in S-Tab.2). Annealing temperature for PCR was standardized at 59°C for 45 sec. Wild type alleles, designated *A1*allele (no duplication) resulted in 278 bp fragment and the variant alleles, designated *A2*allele (with 16 bp duplication) resulted in 294 bp fragment.

Genotypic analysis of the *TP53 Int6 MspI* polymorphism was determined by the PCR-based restriction fragment length polymorphism (PCR–RFLP) method. PCR was performed by amplifying genomic DNA with respective primers (listed in supplemental Table 2). PCR was performed by amplifying genomic DNA with respective primers (listed in supplemental Table 2). Annealing temperature for PCR was standardized at 60°C for 45 sec. Following PCR, PCR products subjected to restriction digestion with *Msp*I (New England Biolabs, Beverey, Massachusetts, United States). The 644bp amplified product was digested overnight with 5 U of *Msp*I at 37°C. The CC genotypes determined by presence of 118, 275, 250 bp bands, AC genotypes determined by presence of 118, 275, 250, 394 bp bands and AA genotypes determined by presence of 394, 250 bp bands due to presence of constant cut site.

Genotypic analysis of the *MDM2 SNP309* (rs2279744) polymorphism was determined by the PCR-based restriction fragment length polymorphism (PCR–RFLP) method. PCR was performed by amplifying genomic DNA with respective primers (listed in supplemental Table 2). Annealing temperature for PCR was standardized at 60°C for 45 sec. Following PCR, PCR products subjected to restriction digestion with *MspA*1I (New England Biolabs, Beverey, Massachusetts, United States). The 237bp amplified product was digested overnight with 5 U of *MspA*1I at 37°C. The wild-type allele “*T”*was identified by the presence of 237bp band, while the mutant allele “*G*” was represented by 189bp and 48bp bands.

Genotypic analysis of *p21 codon31*  polymorphism was determined by the PCR-based restriction fragment length polymorphism (PCR–RFLP) method. PCR was performed by amplifying genomic DNA with respective primers (listed in supplemental Table 2). Annealing temperature for PCR was standardized at 64.5°C for 45 sec. Following PCR, PCR products subjected to restriction digestion with *BsmA*I (New England Biolabs, Beverey, Massachusetts, United States). The 274 bp amplified fragment of exon 2 of the *CDKN1A* gene was produced and digested by the restriction enzyme *BsmA*I 3U (New England Biolabs, Beverey, Massachusetts, United States) at 55°C overnight. The digestion of the wild type allele (*Ser/Ser*) presented a constant site for restriction enzyme recognition and yielded two bands: one of 143 bp and the other of 131 bp. The homozygote genotype for the polymorphic (*Arg/Arg*) allele was characterized by the presence of a second site for the *BsmA*I enzyme, yielding three bands: 131, 75 and 67 bp; and the heterozygote genotype (*Ser/Arg*) showed four bands: 142, 131, 75 and 67 bp. After digestion, the reaction was analyzed by means of electrophoresis on 3% agarose gel at 120V.

Genotypic analysis of the *P73 73bp* deletion polymorphism was determined by the PCR method. PCR was performed by amplifying genomic DNA with respective primers (listed in supplemental Table 2). Annealing temperature for PCR was standardized at 59°C for 45 sec. Wild type alleles, designated *A1*allele (no deletion) resulted in 322 bp fragment and the variant alleles, designated *A2*allele (with 73 bp deletion) resulted in 249 bp fragment.

**Analysis of association between somatic mutations and germline polymorphisms**

**Haplotype Analysis**

We used genotype data for 34 cases (*TP53* mutants) and 59 controls (*TP53* non muatnts) at three SNPs in the gene *TP53* for the haplotype analyses. We determined the haplotype frequencies by Expectation-Maximization (EM) algorithm using the command “haplo.em” implemented in the CRAN R package “haplo.stats”. Then we tested for equality in frequencies of the three haplotypes which have the highest frequencies between cases and controls. We used the large sample test for equality of binomial proportions at the usual level of 0.05.

**SNP-SNP interaction:**

We used two contrasting approaches for validation of the significant findings.

1. Multifactor Dimensionality Reduction (MDR) was performed using the software MDR with all parameters set as default and the threshold as 1.00. We explored for all two dimensional to six dimensional SNP-SNP interactions and the best model was identified for each dimensional interaction by grouping them as “high risk” and “low risk”.

2. Alternatively, we assumed a logistic regression model in which we considered the mutation type as the response and the genotypes of the 6 SNPs as the explanatory variables (coded as 0,1 or 2). The significance of the regression coefficients (at level 0.05) were determined using the command “glm” in R.
