# Supplementary material for: A novel hotspot and rare somatic mutation p.A138V, at TP53 is associated with poor survival of pancreatic ductal and periampullary adenocarcinoma patients
Source: Mol Med. 2020 Jun 17;26:59. doi: 10.1186/s10020-020-00183-1 (PMC7302128; doi:10.1186/s10020-020-00183-1)
Supplement: Supplementary file 7 — Additional file 7. [file 10020_2020_183_MOESM7_ESM.docx]

**Supplementary Table 5: Frequently mutated genes identified in Pancreatic ductal and ampullary adenocarcinoma by simulation using publicly available databases**

|  | **Gene** | **# patient pools (8 patients) without non-silent mutation in reported driver gene** | **# patient pools (8 patients) with non-silent mutation in reported driver gene** |
| --- | --- | --- | --- |
| **Known PDAC (TCGA) drivers** | **KRAS** | 0 | 10000 |
|  | **TP53** | 1 | 9999 |
|  | **SMAD4** | 982 | 9018 |
|  | **CDKN2A** | 2745 | 7255 |
|  | **GNAS** | 5638 | 4362 |
|  | **RNF43** | 5919 | 4081 |
|  | **ARID1A** | 6310 | 3690 |
|  | **TGFBR2** | 6403 | 3597 |
|  | **RREB1** | 6381 | 3619 |
|  | **PBRM1** | 7511 | 2489 |
|  |  |  |  |
| **Known AC drivers** | **KRAS** | 45 | 9955 |
|  | **TP53** | 259 | 9741 |
|  | **APC** | 310 | 9690 |
|  | **ELF3** | 3020 | 6980 |
|  | **SMAD4** | 2939 | 7061 |
|  | **CTNNB1** | 2949 | 7051 |
|  | **MUC4** | 1193 | 8807 |
