# Supplementary material for: A novel hotspot and rare somatic mutation p.A138V, at TP53 is associated with poor survival of pancreatic ductal and periampullary adenocarcinoma patients
Source: Mol Med. 2020 Jun 17;26:59. doi: 10.1186/s10020-020-00183-1 (PMC7302128; doi:10.1186/s10020-020-00183-1)
Supplement: Supplementary file 9 — Additional file 9. [file 10020_2020_183_MOESM9_ESM.docx]

**Supplemental Table 7: Statistical analysis of the protein DNA docking result obtained by HADDOCK**

| Parameter description | Wt/  A138V | HADDOCK score | Cluster size | RMSD | VDW energy | Electrostatic energy | Desolvation energy | Restraints violation energy | Buried Surface Area | Z-Score |
| --- | --- | --- | --- | --- | --- | --- | --- | --- | --- | --- |
| All Hydrogen atoms are involved in calculation. Solvent Docking included but Water interaction analysis excluded | Wt | -192.5 +/- 7.2 | 6 | 7.2 +/- 0.6 | -51.4 +/- 5.8 | -422.3 +/- 48.8 | 35.2 +/- 1.3 | 396.9 +/- 114.98 | 1441.5 +/- 92.2 | -1.8 |
|  | A138V | -208.0 +/- 18.6 | 8 | 13.6 +/- 0.1 | -65.1 +/- 5.2 | -468.4 +/- 37.9 | 36.3 +/- 2.0 | 555.9 +/- 54.72 | 1656.5 +/- 43.2 | -1.9 |
| All Hydrogen atoms are involved in calculation. Solvent Docking and Water interaction included | Wt | -190.3 +/- 10.7 | 17 | 18.0 +/- 0.7 | -56.9 +/- 6.7 | -397.7 +/- 39.0 | 25.7 +/- 2.1 | 626.3 +/- 78.00 | 1566.6 +/- 109.1 | -1.9 |
|  | A138V | -190.8 +/- 9.6 | 11 | 13.7 +/- 0.3 | -66.2 +/- 5.0 | -419.8 +/- 46.9 | 35.0 +/- 1.3 | 611.2 +/- 64.98 | 1639.2 +/- 16.8 | -1.7 |
| All Hydrogen atoms are involved in calculation. Solvent Docking and Water interaction included with explicit Desolvation energy calculation. | Wt | -11660.5 +/- 4.7 | 16 | 19.3 +/- 0.1 | -38.4 +/- 7.1 | -402.9 +/- 18.9 | -11619.2 +/- 10.8 | 776.2 +/- 57.47 | 1459.6 +/- 90.1 | -1.9 |
|  | A138V | -11538.8 +/- 31.7 | 7 | 7.3 +/- 0.6 | -25.9 +/- 5.0 | -321.7 +/- 26.5 | -11507.7 +/- 25.8 | 592.0 +/- 29.49 | 1230.9 +/- 78.8 | -1.9 |

|  |  |  |
| --- | --- | --- |
|  |  |  |
|  |  |  |
|  |  |  |
|  |  |  |
|  |  |  |
|  |  |  |
|  |  |  |
|  |  |  |
|  | |  |
|  | |  |
|  | |  |
|  | |  |
|  | |  |
