# Supplementary material for: A novel hotspot and rare somatic mutation p.A138V, at TP53 is associated with poor survival of pancreatic ductal and periampullary adenocarcinoma patients
Source: Mol Med. 2020 Jun 17;26:59. doi: 10.1186/s10020-020-00183-1 (PMC7302128; doi:10.1186/s10020-020-00183-1)
Supplement: Supplementary file 11 — Additional file 11. [file 10020_2020_183_MOESM11_ESM.docx]

**Supplimental Table 9: Haplotype frequency between TP53 mutant and non mutant group**

| Haplotype No. | *TP53* rs1042522 (R/P) | *TP53* PIN3 16bp (I/D) | *TP53* rs1625895 A/C | Frequency of *TP53* mutant group | Frequency of TP53 non mutant group | P Value |
| --- | --- | --- | --- | --- | --- | --- |
| 1. | R | D | C | 0.53 | 0.45 | 0.48 |
| 2. | P | D | C | 0.23 | 0.30 | 0.51 |
| 3. | P | I | A | 0.17 | 0.14 | 0.49 |
| 4. | P | D | A | 0.03 | 0.009 | - |
| 5. | P | I | C | 0.01 | 0.02 | - |
| 6. | R | I | C | 0.01 | 0.03 | - |
